# Structured water molecules drive activation and G protein selectivity in the GPR174 receptor

**DOI:** 10.1371/journal.pbio.3003447

**Published:** 2026-05-07

**Authors:** Ying-Jun Dong, Kun Xi, Ya-Zhi Zhang, Jian-Heng Xue, Dan-Dan Shen, Shao-Kun Zang, Ruozhu Zhao, Hai Qi, Chunyou Mao, Wei-Wei Wang, Yan Zhang

**Affiliations:** 1 Department of Pathology of Sir Run Run Shaw Hospital, Department of Pharmacology, MOE Frontier Science Center for Brain Research and Brain-Machine Integration, and Liangzhu Laboratory, Zhejiang University School of Medicine, Hangzhou, China; 2 School of Pharmacy, Hangzhou Medical College, Hangzhou, China; 3 Department of Cardiology of Sir Run Run Shaw Hospital, Zhejiang University School of Medicine, Hangzhou, China; 4 Center for Structural Pharmacology and Therapeutics Development, Sir Run Run Shaw Hospital, Zhejiang University School of Medicine, Hangzhou, China; 5 Department of General Surgery, Sir Run Run Shaw Hospital, Zhejiang University School of Medicine, Hangzhou, China; 6 School of Basic Medical Sciences, Tsinghua Medicine, Tsinghua University, Beijing, China; University of Zurich, SWITZERLAND

## Abstract

G protein-coupled receptor 174 (GPR174), a key modulator of autoimmune responses, maintains immune homeostasis through distinct G protein signaling pathways, particularly G_s_ and G_i_. Although the structural mechanism of lysophosphatidylserine (LysoPS)-activated GPR174 in the G_s_ pathway has been characterized, how hydration-mediated interactions influence GPR174 activation and signaling selectivity remains unclear. Here, we determined high-resolution cryo-electron microscopy (cryo-EM) structures of LysoPS-activated human GPR174 bound to G_s_ (2.0 Å) and G_i_ (3.4 Å), revealing a continuous hydration-mediated signal transduction network that bridges the sodium-binding pocket, the NPxxY and DRY motifs, and the G protein-binding interface. This network stabilizes the active-state conformation of GPR174 and dynamically reshapes the intracellular cavity, thereby enabling differential engagement of G_s_ and G_i_. Molecular dynamics simulations and functional assays demonstrated that the hydration network is essential for receptor activation and selectively modulates G protein coupling. To evaluate its conservation, we performed sequence alignments and structural analyses across class A GPCRs, defining three hydration cavities: the conserved water cavity (CWC), the junctional water cavity (JWC), and the extended water cavity (EWC), whose hydration is determined by residue properties at position 5.58. Together, our study reveals a hydration-driven molecular mechanism that underlies the activation of GPR174 and its dual G protein selectivity. These findings advance the understanding of hydration-mediated signaling in GPR174 and provide a framework for investigating water-mediated regulation across class A GPCRs.

## Abstract 2

The GPR174 receptor modulates immune homeostasis by engaging distinct G protein pathways, but how hydration-mediated interactions influence its activation remains unclear. This study shows that these networks stabilize the active-state conformation of GPR174 and reshape its intracellular cavity by enabling selectivity.

## Introduction

G protein-coupled receptors (GPCRs) are versatile transmembrane sensors that transduce extracellular signals into intracellular responses via conformational activation and selective G protein coupling [1–6]. This process is coordinated by conserved microswitches such as the DRY and NPxxY motifs, along with dynamic hydration and electrostatic rearrangements within the transmembrane core [7–9]. Recent studies have implicated hydration-mediated interactions as key allosteric regulators of receptor activation, with conserved hydration networks shown to rearrange between inactive and active states [5,6,10–12]. However, the organization of internal water networks, the identity of conserved and variable sites, and how these features may influence G protein selectivity remain unclear.

**Fig 1:**
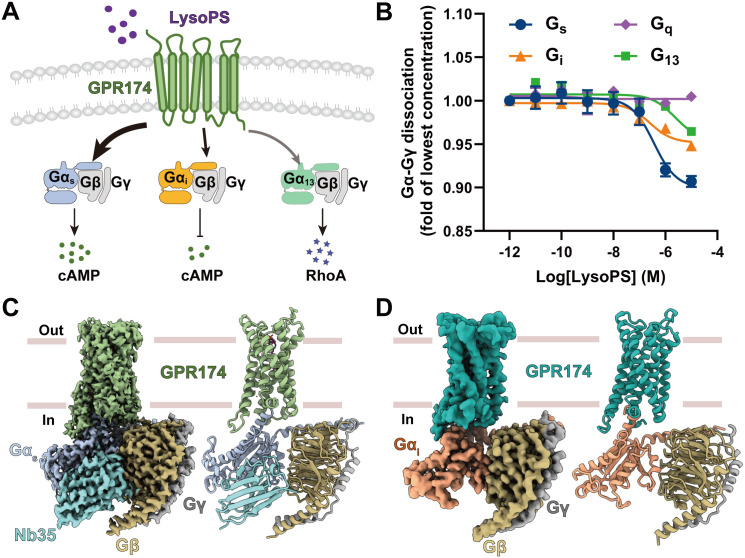
Signaling profiles and structural characterization of GPR174. **(A)** Schematic overview of GPR174 signaling activated by LysoPS. **(B)** Dose-response curve of GPR174 activation by LysoPS (18:0), as measured by the NanoBiT G protein dissociation assay. Data are presented as mean ± SEM from at least three independent experiments, each performed in triplicate. The data used to generate graphs in Fig 1B is available in S1 Data. **(C)** Cryo-EM density map and structural model of LysoPS (18:0)-bound GPR174-G_s_ complex. Pale green, GPR174; steel blue, Gα_s_; goldenrod, Gβ; dark gray, Gγ; dark cyan, Nb35. **(D)** Cryo-EM density map and structural model of the GPR174-G_i_ complex. Teal, GPR174; coral, Gα_i_; goldenrod, Gβ; dark gray, Gγ.

GPR174, a class A GPCR, is predominantly expressed in lymphoid tissues and genetically associated with autoimmune disorders such as Graves’ disease and Addison’s disease [13–15]. GPR174 maintains immune homeostasis by coupling to multiple G proteins and exhibits context-dependent engagement of G_s_ and G_i_ in T and B lymphocytes [16–21]. Studies have shown that G_s_ signaling promotes T cell exhaustion and immune suppression, whereas G_i_ signaling enhances effector T cell differentiation and cytotoxic function by lowering intracellular cAMP [22]. Using functional assays, we found that LysoPS stimulation of GPR174 not only robustly activates the G_s_ pathway but also induces G_i_ signaling, with no detectable response through G_q_ and weak G_13_ coupling (Fig 1A and 1B) [20,23–26]. Despite this functional divergence, the mechanism underlying how GPR174 selectively regulates these pathways remains unclear. To address these questions, we determined high-resolution cryo-electron microscopy (cryo-EM) structures of LysoPS-bound human GPR174 in complex with G_s_ (2.0 Å) and G_i_ (3.4 Å) (Fig 1C and 1D), revealing a continuous hydration-mediated network that links the sodium-binding pocket, the NPxxY and DRY motifs, and the intracellular G protein-binding interface. Mutagenesis of water-coordinating residues disrupted hydrogen-bond connectivity and impaired activation, confirming the functional relevance of this network. Molecular dynamics simulations further showed prolonged hydrogen-bond lifetimes, consistent with stable structural waters that support G protein binding. We also defined three hydration cavities across class A GPCRs: the conserved water cavity (CWC), the junctional water cavity (JWC), and the extended water cavity (EWC). Sequence and cavity volume analyses showed that the CWC and JWC are structurally preserved across class A GPCRs, whereas position 5.58 is the primary determinant of EWC hydration. Structural and functional comparisons further supported that hydration networks contribute to the selective coupling of GPR174 to G_s_ and G_i_ proteins, identifying water molecules as integral determinants of signaling specificity. These findings advance our understanding of hydration-mediated signaling and provide a framework for investigating water-mediated regulation across class A GPCRs.

## Results

### Signaling profiles and structural characterization of GPR174

To investigate the signaling profiles of GPR174 in response to LysoPS, we employed NanoLuc Binary Technology (NanoBiT), a robust method for quantifying the engagement of multiple G proteins by GPCRs [23,27]. We observed that LysoPS activation of GPR174 exhibits a strong coupling preference for G_s_ over G_i_, with no detectable response through G_q_ and a weak response through G_13_ (Fig 1A and 1B; S1 and S2 Tables). These results indicate that LysoPS activates G_s_, consistent with previous pharmacological studies [24,28]. Notably, the significantly stronger signaling through the G_s_ pathway compared to G_i_ supported efficient assembly and structural determination of the GPR174-G_s_ complex [20,26,29]. To further characterize the structural basis of LysoPS-triggered activation and G protein selectivity, we applied the same NanoBiT tethering strategy to assemble both GPR174-G_s_ and GPR174-G_i_ complexes [27]. Finally, the structures of LysoPS-bound GPR174-G_s_ and GPR174-G_i_ complexes were solved at resolutions of 2.0 Å and 3.4 Å, respectively (Figs 1C, 1D, S1, and S2; S3 Table). These high-resolution density maps enabled clear visualization of LysoPS binding in the GPR174-G_s_ complex and the overall architecture of the seven-transmembrane domain of GPR174 in both G_s_- and G_i_-bound structures.

### Identification of hydration-mediated network in GPR174

**Fig 2:**
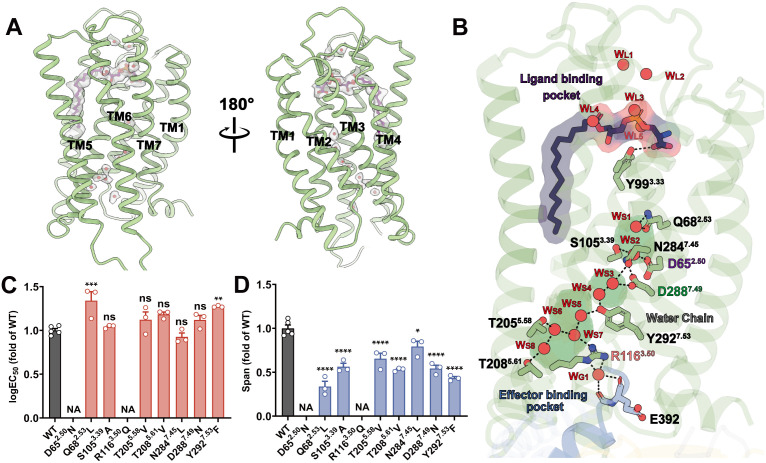
Hydration-mediated signaling network of the GPR174-G_s_ complex. **(A)** The overall structure of GPR174 with the LysoPS ligand (purple), with ordered internal water molecules shown as red spheres. **(B)** Structural depiction of the hydration-mediated signaling network in the GPR174-G_s_ complex. Hydrogen bonds are shown as black dashed lines; hydration-associated residues are depicted as pale green sticks. **(C)** Effects of mutations at key residues involved in the hydration-mediated signaling network on the logEC_50_ of LysoPS (18:0)-induced Gα_s_-Gγ dissociation. Values are mean ± SEM from independent experiments (*n* ≥ 3), each performed in triplicate. Exact n is indicated on the panel. NA, not applicable; ns, *P* > 0.05; *P < 0.05; **P < 0.01; ***P < 0.001; ****P < 0.0001. Statistical significance was assessed using one-way ANOVA followed by Dunnett’s multiple comparisons test vs. WT. Statistical analysis results are summarized in S4 Table. **(D)** Effects of mutations at key residues involved in the hydration-mediated signaling network on the span of LysoPS (18:0)-induced Gα_s_-Gγ dissociation. Values are mean ± SEM from independent experiments (*n* ≥ 3), each performed in triplicate. Exact *n* is indicated on the panel. NA, not applicable; ns, *P* > 0.05; *P < 0.05; **P < 0.01; ***P < 0.001; ****P < 0.0001. Statistical significance was assessed using one-way ANOVA followed by Dunnett’s multiple comparisons test vs. WT. Statistical analysis results are summarized in S4 Table. The data used to generate graphs in Fig 2C and 2D are available in S1 Data.

The high-resolution cryo-EM structure of the LysoPS (18:0)-bound GPR174-G_s_ complex reveals a well-defined internal hydration network comprising 14 water molecules, which are primarily distributed across three functional regions: (i) five water molecules (W_L1_–W_L5_) located in the orthosteric binding pocket stabilize the polar headgroup of LysoPS, initiating signal transduction; (ii) eight water molecules (W_S1_–W_S8_) participate in the formation and stabilization of key activation motifs, including the canonical DRY and NPxxY motifs, as well as other less conserved elements; (iii) a single water molecule (W_G1_) mediates an interaction network at the interface between the intracellular pocket of GPR174 and the C-terminal α5 helix of Gα_s_ (Figs 2A, 2B, and S3A).

The orthosteric pocket of GPR174, framed by ECL2 and the extracellular halves of TM1-TM7, exhibits an amphipathic organization comprising a polar headgroup-binding region and a distal hydrophobic groove. Five structured waters stabilize the zwitterionic headgroup of LysoPS, facilitating conformational priming for receptor activation (Fig 2B). The lipid tail of LysoPS is embedded in a deep hydrophobic valley formed by TM3, TM4, TM5, and TM6, where it is stabilized by aromatic stacking interactions with Y99^3.33^, Y103^3.37^, F152^4.50^, Y246^6.51^, and F250^6.55^ (S3B and S3C Fig). This hydrophobic subpocket contributes to ligand anchoring and defines the spatial boundary of the polar hydration cluster.

On the intracellular side, a structured hydration-mediated signaling network, composed of W_S1_-W_S8_ and W_G1_, forms a hydrogen-bond “necklace” that bridges the sodium ion-binding pocket, the NPxxY motif, the DRY motif, and the α5 helix of Gα_s_ (Fig 2B). This network can be subdivided into three clusters. The first cluster comprises W_S1_ and W_S2_ in the sodium-binding pocket, where they form hydrogen bonds with D65^2.50^, S105^3.39^, Q68^2.53^, and N284^7.45^. Interactions of N284^7.45^ and D288^7.49^ with W_S2_ and W_S3_ provide a link between the first and second clusters. The second cluster consists of W_S3_ and W_S4_ adjacent to the NPxxY motif, where N284^7.45^ and Y292^7.53^ act as coordinating residues. Y292^7.53^ further engages W_S4_ and W_S5_, extending the network toward the third cluster. The third cluster, formed by W_S5_-W_S8_ proximal to the DRY motif, connects R116^3.50^ with T205^5.58^ and T208^5.61^, propagating the hydration pathway from the NPxxY motif to the cytoplasmic end of TM5. Collectively, these three clusters establish a continuous water-mediated pathway through the receptor core (S3D–S3F Fig). Through these contacts, W_G1_ contributes to both stabilizing the active-state conformation of GPR174 and anchoring the Gα_s_ protein (S3G Fig). This structured hydration network reinforces the active-state conformation and supports G protein engagement.

To assess the functional relevance of the structured hydration network resolved in the cryo-EM structure, we mutated polar residues that coordinate internal water molecules along the W_S1_-W_S8_ and W_G1_ pathway (Fig 2C and 2D; S4 Table). Two key residues, D65^2.50^ and R116^3.50^, are centrally positioned within the hydrogen-bonded network. D65^2.50^ coordinates W_S2_ near the receptor core, whereas R116^3.50^ interacts with W_S6_ and W_G1_ at the G protein interface. Substitution of D65^2.50^ with asparagine and R116^3.50^ with glutamine replaced charged side chains with uncharged polar groups and severely impaired LysoPS-induced receptor activity. We next tested D288^7.49^ and Y292^7.53^, two conserved residues within the NPxxY motif. In the cryo-EM structure, D288^7.49^ coordinates W_S2_ and W_S3_, and Y292^7.53^ interacts with W_S4_ and W_S5_. Substitution of either residue reduced LysoPS-induced receptor activity. We also evaluated additional water-coordinating residues by introducing hydrophobic substitutions that preserve side chain volume while disrupting hydrogen bonding. These included Q68^2.53^L, S105^3.39^A, T205^5.58^V, T208^5.61^V, and N284^7.45^L. Each mutation resulted in reduced receptor activity, consistent with a role in stabilizing internal waters and maintaining the geometry of the hydrogen-bond network.

To investigate the dynamic stability of the signaling-associated hydration network, we further performed molecular dynamics simulations specifically focused on nine internal waters (W_S1_–W_S8_ and W_G1_) in GPR174. These water molecules exhibit prolonged residence times (0.19–1.34 ns), far exceeding the ~10 ps exchange rate of bulk solvent and approaching that of deeply buried structural waters reported in other class A GPCRs (S4A Fig). Upon removal and rehydration, these waters spontaneously returned to their original positions within 50 ns and reformed the hydrogen-bonding network with lifetimes comparable to the native configuration (S4B Fig). RMSD analysis revealed that coordinating residues, particularly those within the DRY and NPxxY motifs, remained conformationally stable during 1000-ns simulations (RMSD < 3 Å), reinforcing the structural and functional significance of the hydration network (S4C–S4E Fig; S5 and S6 Tables). Our structural comparison of GPR174 in complexes with both G_s_ and G_i_ proteins reveals a high similarity in the positions of key residues along the hydration network (average RMSD < 0.6 Å). In the GPR174-G_i_ complex, the G_i_ simulations exhibited shorter water-residue hydrogen-bond lifetimes relative to G_s_ (S5A Fig and S7 Table). The overall complex remained stable across three independent 1 µs trajectories (S5B Fig). These simulations further showed rapid reoccupation of the internal hydration sites (W_S1_–W_S8_ and W_G1_) within ~50 ns (S5C Fig). Consistent with the reduced hydrogen-bond lifetimes, coordinating residues displayed increased RMSD values in the G_i_ simulations relative to G_s_ (S5D Fig), which likely reduces the visibility of internal waters at 3.4 Å resolution in the current reconstruction. Consistent with this, the same set of hydration-network mutations also reduced LysoPS-induced G_i_ signaling (S5E and S5F Fig; S8 and S9 Tables).

Together, these results support a model in which the W_S1_–W_S8_ and W_G1_ network in GPR174 forms a continuous hydration network linking conserved activation motifs to the G protein interface, reinforcing the active-state conformation and enabling intracellular signal propagation.

### Hydration cavities and allosteric water networks across class A GPCRs

**Fig 3:**
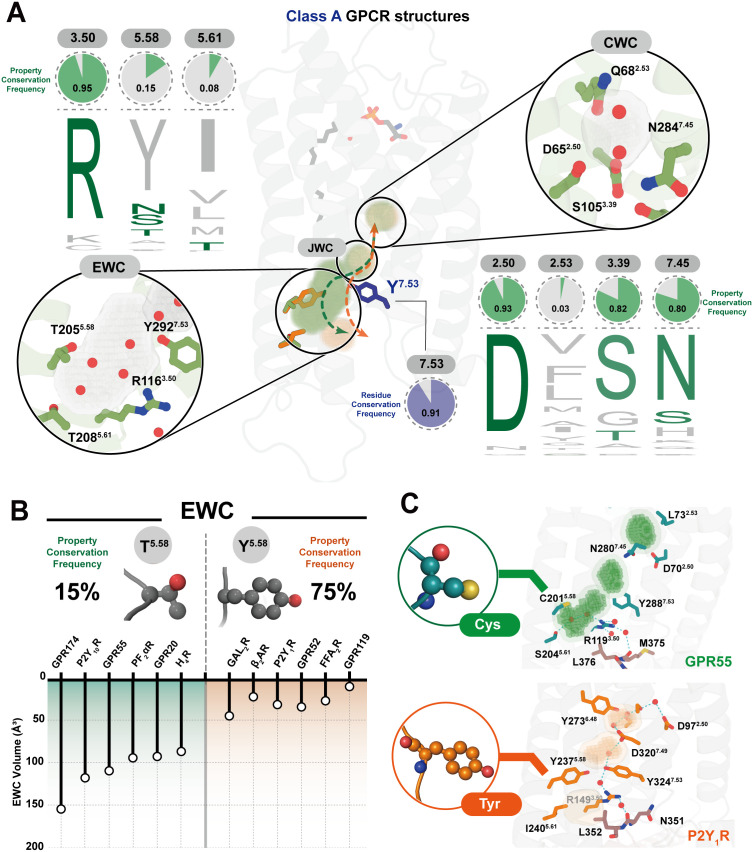
Conserved and divergent hydration cavities across class A GPCRs. **(A)** Sequence conservation analysis of residues forming three water-associated cavities: conserved water cavity (CWC), junctional water cavity (JWC), and extended water cavity (EWC). Logos indicate residue frequency at positions 2.50, 2.53, 3.39, 3.50, 5.58, 5.61, 7.45, and 7.53 across class A GPCRs. Green pie charts represent the property conservation frequency of corresponding residues in GPR174. Insets show examples of polar interactions within these cavities observed in GPR174 (green) and P2Y_1_R (orange, PDB ID: 7XXH), with cavity regions identified by parKVFinder and visualized in PyMOL. **(B)** Distribution and functional impact of residue properties at position 5.58. Across class A GPCRs, this position is occupied mainly by either short side-chain polar uncharged residues (e.g., Ser, Thr, Cys) or by bulky hydrophobic/aromatic residues (e.g., Tyr, Phe). To assess structural consequences, EWC volumes were quantified from available active-state structures, including GPR174 (this study), P2Y_10_R (PDB ID: 8KGG), GPR55 (PDB ID: 9GE2), PF_2_αR (PDB ID: 8IUK), GPR20 (PDB ID: 8HS3), H_4_R (PDB ID: 7YFC), GAL_2_R (PDB ID: 7WQ4), β_2_AR (PDB ID: 3SN6), P2Y_1_R (PDB ID: 7XXH), GPR52 (PDB ID: 6LI3), FFA_2_R (PDB ID: 8J24), and GPR119 (PDB ID: 7WCM). Receptors with short side-chain polar uncharged residues generally maintain enlarged EWCs, whereas those with bulky hydrophobic/aromatic residues exhibit restricted cavities. **(C)** Structural comparison of GPR55 (C^5.58^, PDB ID: 9GE2) and P2Y_1_R (Y^5.58^, PDB ID: 7XXH). In GPR55, ordered water molecules are observed in the EWC based on cryo-EM density. In P2Y_1_R, water occupancy detected by MD simulations is limited to the CWC and JWC.

While GPR174 illustrates how ordered internal water molecules can form a functional hydration network, the presence and conservation of such networks across class A GPCRs are not well established. We therefore sought to define the structural basis for hydration compatibility across receptors by analyzing water-accessible cavities that may facilitate internal water retention. To investigate the conservation of internal hydration features across class A GPCRs, we first defined three principal water-enriched cavities in GPR174 based on the cryo-EM structure and parKVFinder analysis (Fig 3A) [30]. These include (i) the CWC, located proximal to the sodium-binding pocket and shaped primarily by residues 2.50, 3.39, and 7.45; (ii) the JWC, situated adjacent to Y^7.53^ and linking conserved motifs; and (iii) the EWC, defined by surrounding residues 3.50, 5.58, and 5.61.

To assess the conservation of hydration-associated cavities, we performed sequence alignments of residues involved in shaping the CWC, JWC, and EWC across class A GPCRs using GPCRdb [31]. For each cavity, we calculated the frequency of residues with physicochemically similar side chain properties to those observed in GPR174 (Fig 3A). Among the residues shaping the CWC, positions 2.50, 3.39, and 7.45 are highly conserved. Over 80% of class A GPCRs contain residues with physicochemically similar side chains at these positions. In contrast, position 2.53 exhibits low conservation, suggesting that it may play a modulatory role in determining the volume of the CWC. We next examined the sequence profile of the JWC, which is located adjacent to Y^7.53^. Tyrosine is conserved at position 7.53 in 91% of class A GPCRs, which suggests that the position and geometry of the JWC may be structurally preserved across these receptors. We then analyzed the EWC, which is defined by residues 3.50, 5.58, and 5.61, located close to the cytoplasmic end of TM5 and TM6. Among these residues, R^3.50^ is highly conserved, present in over 90% of class A GPCRs. In contrast, positions 5.58 and 5.61 show marked variability. Position 5.58, which represents the core determinant of the EWC and is the primary driver of cavity formation, falls into two distinct categories: small, uncharged polar residues (e.g., Asn, Thr, Cys) are observed in roughly 15% of receptors, whereas bulky aromatic residues (e.g., Tyr, Phe) occupy this position in approximately 75% of receptors, imposing steric constraints that limit hydration (Fig 3B). By contrast, 5.61 lies at the periphery of the cavity and plays a minor role in shaping the EWC. This distribution suggests that the key regulatory position 5.58 governs the size and hydration capacity of the EWC. Receptors with small, uncharged polar residues at this site allow water molecules to occupy the cavity and substitute for side-chain packing, whereas those with bulky aromatic residues provide steric support during TM6 outward movement.

To evaluate whether side-chain composition at cavity-facing residues correlates with cavity size, we performed cavity volume measurements across a panel of class A GPCRs (S6 and S7 Figs; S10 Table). CWC and JWC volumes remained relatively stable across receptors, consistent with the peripheral position of 2.53 relative to the sodium-binding pocket and the high conservation of Y^7.53^ (S8 Fig). In contrast, the EWC volume exhibits a marked dependence on the residue at position 5.58 (Fig 3B). Receptors with small polar residues at 5.58 (e.g., GPR174, P2Y_10_R, GPR55) consistently exhibit significantly larger EWC volumes compared to those with bulky hydrophobic residues (e.g., GAL_2_R, β_2_AR, P2Y_1_R) [25,32–41], consistent with our sequence-based analysis. To further validate the key regulatory role of residue composition at position 5.58 in modulating EWC volume, we focused on two representative receptors, GPR55 and P2Y_1_R, which feature distinct side-chain properties at this position (Fig 3C). In GPR55, where residue 5.58 is cysteine, two water molecules are resolved in the EWC of the active-state GPR55-G_13_ structure determined by cryo-EM [32]. In P2Y_1_R, where residue 5.58 is tyrosine, we analyzed the active-state cryo-EM structure of the P2Y_1_R-G_11_ complex and performed molecular dynamics simulations, which did not reveal the presence of water molecules in the EWC [38]. These observations indicate that the side chain properties at 5.58 play a central role in modulating EWC architecture and hydration.

Collectively, these results define a modular hydration network in class A GPCRs, where conserved cavities (CWC, JWC) stabilize core activation motifs, whereas the EWC provides receptor-specific flexibility. Position 5.58 plays a key role, where small, uncharged polar residues permit water molecules to occupy the cavity and act as a scaffold, while bulky residues substitute for this role by providing steric support. Thus, TM6 outward movement enlarges the TM5-TM6 cavity, which is stabilized either by water-mediated scaffolding or by bulky residues at 5.58, thereby maintaining the active conformation and promoting signaling. To further provide functional support for this conserved hydration-linked transmission scaffold, we quantified cAMP signaling output in P2Y_1_R mutants at structurally equivalent positions, which showed that mutations at these positions reduced signaling efficiency relative to WT (S9 Fig; S11 and S12 Tables).

### Structural and hydration determinants of G protein selectivity in GPR174

More than half of GPCRs are capable of engaging either G_s_ or G_i_ proteins, and a subset exhibits bifunctional coupling to both subtypes, as observed in GPR4, GPR120, CCK_1_R, 5-HT_4_R, and GCGR [3,42–45]. GPR174 couples to G_s_, G_i_, and the G_13_ family of G proteins, with preferential activation of G_s_ over G_i_ signaling (Fig 1A) [43].

We determined cryo-EM structures of GPR174 in complex with both G_s_ and G_i_, which enabled a comparative analysis of structural features underlying G protein selectivity. The receptor adopts a highly similar overall conformation in both states (Cα RMSD 0.77 Å; S10A and S10B Fig), and the orthosteric binding pocket remains nearly identical (all-atom RMSD < 0.5 Å; S10C Fig). The G_i_ complex was assembled in the presence of LysoPS, but the pocket density was insufficient for reliable ligand modeling, and the final G_i_ coordinates were deposited without a modeled ligand. Alanine substitutions of ligand-interacting residues impaired both G_s_- and G_i_-mediated signaling under our assay conditions (S10D–S10G Fig and S13–S16 Tables), indicating that these residues are involved in receptor activation and are unlikely to be the primary determinants of G protein subtype selectivity.

**Fig 4:**
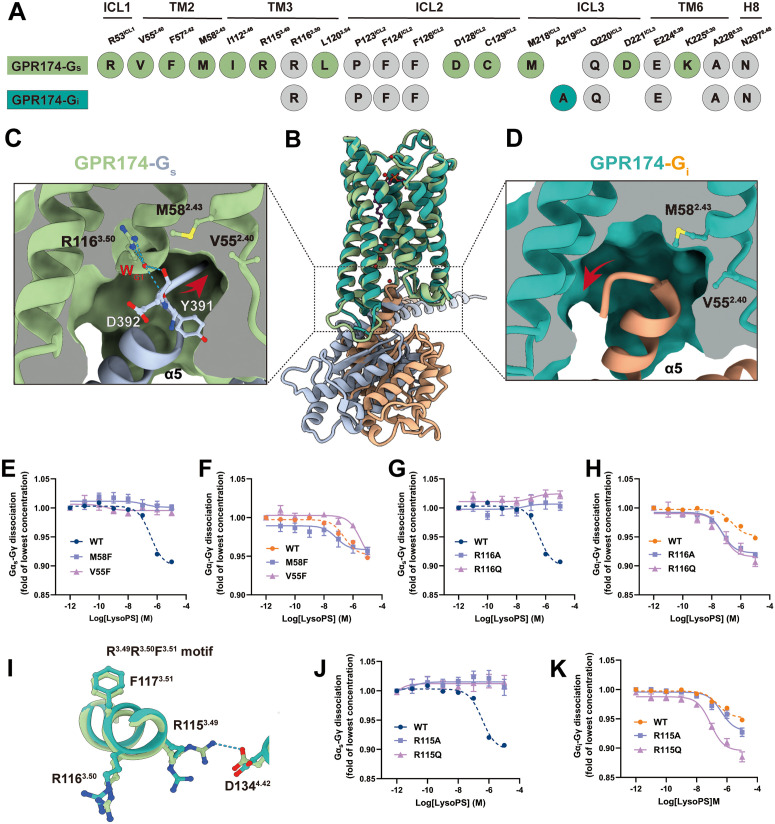
Hydration-mediated structural mechanisms underlying G protein selectivity in GPR174. **(A)** Residues involved in selective interactions with Gα_s_ (pale green circles) or Gα_i_ (teal circles) at the receptor-G protein interface. Residues shared between Gα_s_ and Gα_i_ interfaces are shown in gray. **(B)** Side view of superposed GPR174-G_s_ (pale green) and GPR174-G_i_ (teal) complexes. **(C) and (D)** Conformational details of the α5-helix upon GPR174 coupling with G_s_ (C, light steel blue) or G_i_ (D, light salmon). **(E) and (F)** LysoPS (18:0) concentration-response curves for GPR174 activation via G_s_ (E) and via G_i_ (F), shown for WT as well as the hydrophobic-cavity mutants V55^2.40^F and M58^2.43^F. **(G) and (H)** LysoPS (18:0) concentration-response curves for GPR174 activation via G_s_ (G) and via G_i_ (H), shown for WT as well as the R116^3.50^ mutants R116^3.50^A and R116^3.50^Q. **(I)** Structural comparison of the DRY motif region in GPR174-Gα_s_ (pale green) or GPR174-Gα_i_ (teal) complexes. **(J) and (K)** LysoPS (18:0) concentration–response curves for GPR174 activation via G_s_ (J) and via G_i_ (K), shown for WT as well as the DRY-motif mutants R115^3.49^A and R115^3.49^Q. For all functional curve panels, data are presented as mean ± SEM from at least three independent experiments, each performed in triplicate, and fitted potency and response parameters are reported in S13–S16 Tables. The data used to generate graphs in Fig 4E–4H, 4J, and 4K are available in S1 Data.

In contrast, the G_s_ complex features an enlarged G protein-binding interface, increasing from 890 Å^2^ in the G_i_-coupled complex to 1,560 Å^2^ in the G_s_-coupled complex, and involves significantly more contact residues (Figs 4A and S10H). To assess the functional relevance of these contact residues, we performed alanine substitutions at multiple interface positions. Most of these mutations impaired G_s_ or G_i_ signaling, indicating that both interfaces rely on extensive polar and nonpolar contacts for productive G protein engagement (S10I–S10L Fig and S13–S16 Tables). These structural differences are associated with distinct configurations of the G protein α5 helix. In the G_s_ complex, the α5 helix of Gα_s_ inserts deeply into a hydrophobic pocket formed by TM2 and TM3, whereas in the G_i_ complex, the α5 helix remains more surface-exposed and does not occupy this cavity (Fig 4B). Within this context, three features contribute to the selectivity of G_s_ engagement. First, in the G_s_ complex, the α5 helix of Gα_s_ inserts into a hydrophobic cavity formed by TM2 and TM3 (V55^2.40^, M58^2.43^, Y293^7.54^, F299^8.50^), where it is stabilized by extensive van der Waals interactions (Fig 4C). The α5 helix of Gα_i_ adopts a more surface-exposed configuration and does not engage this hydrophobic pocket (Fig 4D). Mutations that introduced bulky side chains into this cavity (V55^2.40^F/M58^2.43^F) abolished G_s_ signaling and produced a much weaker effect on G_i_ coupling, which remained measurable in our assay (Fig 4E and 4F; S13–S16 Tables), indicating that this site is critical for G_s_ engagement. Second, a structured water molecule (W_G1_), resolved only in the G_s_-bound complex, forms a hydrogen-bond bridge between R116^3.50^ (of the conserved DRY motif) and the backbone carbonyls of Y391^H5.23^ and D392^H5.24^ on the α5 helix (Fig 4C and 4D). Substitution of R116^3.50^ with glutamine or alanine attenuated G_s_ coupling and exerted a more modest impact on G_i_ coupling (Fig 4G and 4H; S13–S16 Tables). Third, a salt bridge between R115^3.49^ and D134^4.42^, which is not present in the G_i_-bound complex, stabilizes the active-state conformation in the G_s_ structure (Fig 4I). Disruption of this interaction through R115^3.49^A or R115^3.49^Q mutations substantially reduced G_s_ signaling while enhancing G_i_ activity (Fig 4J and 4K; S13–S16 Tables), suggesting that this interaction plays a dual role by promoting G_s_ selectivity and simultaneously constraining G_i_ activation.

These findings indicate that GPR174 achieves G protein selectivity primarily through conformational and hydration-mediated mechanisms, rather than ligand recognition. The combined contributions of the hydrophobic cavity, the water-mediated bridge involving W_G1_ at the DRY motif, and the R115^3.49^ – D134^4.42^ salt bridge promote G_s_-specific engagement while permitting conditional coupling to G_i_.

## Discussion

GPR174 plays an important role in immune regulation through its selective coupling to distinct G proteins. Our structural, functional, and computational analyses converge to suggest a hydration-mediated signaling mechanism that stabilizes the active conformation of GPR174 and contributes to G protein selectivity. By resolving a high-resolution cryo-EM structure of the GPR174-G_s_ complex, we captured long-residence water molecules bridging conserved motifs to the intracellular G protein-binding interface (Fig 2B). Mutagenesis validated the essential role of these water-coordinating residues in supporting receptor activation and G protein selectivity. MD simulations further indicate the presence of a stable receptor-spanning hydrogen-bond chain formed by these waters (Figs 2C, 2D, S5E, and S5F). These findings support the emerging view that water molecules serve functional roles rather than passively filling internal cavities. This concept is further reinforced by studies in rhodopsin and β_2_AR, where structured hydration networks were shown to stabilize activation motifs and dynamically couple to conformational transitions [5,10–12,46].

To generalize these observations, we established a cavity-based framework for describing the internal water network. Three water-enriched cavities were identified: the CWC proximal to the sodium pocket (positions 2.50, 3.39, 7.45), the JWC adjacent to Y^7.53^ linking activation motifs, and the EWC close to the cytoplasmic ends of TM5 and TM6 (positions 3.50, 5.58, 5.61) (Fig 3A). Through sequence alignments and structural analyses, we found that core residues shaping the CWC and JWC are highly conserved across class A GPCRs, consistent with their role in stabilizing key activation motifs. In contrast, the EWC shows marked variability in geometry, driven largely by the side chain properties at position 5.58. Cavity volume measurements across multiple receptors, together with sequence alignments, demonstrated that position 5.58 can accommodate two distinct categories of residues (Fig 3B). Residues with small polar side chains at 5.58 allow a larger, water-filled EWC, whereas bulky hydrophobic residues restrict the cavity volume and limit water occupancy. The active-state structure of CCK_1_R (2.0 Å) further supports this principle, as its bulky residue Y^5.58^ prevents water entry, leaving internal waters only near the NPxxY motif [47]. Overall, these results highlight position 5.58 as a key determinant of EWC hydration. As TM6 moves outward during activation, the TM5–TM6 cavity enlarges. This cavity can be stabilized by either water-mediated scaffolding or bulky side chains at position 5.58, thereby supporting the active conformation and promoting signaling.

Beyond stabilizing activation motifs, hydration also contributes to selective G protein engagement. In the GPR174-G_s_ structure, we identified a terminal water bridge connecting R116^3.50^ in the DRY motif to the α5 helix of Gα_s_ (Fig 4C). This feature was not observed in the G_i_ complex (Fig 4D). In addition, a salt bridge between R115^3.49^ and D134^4.42^ (Fig 4I), together with a hydrophobic pocket formed by M58^2.43^ and V55^2.40^, further shapes the intracellular interface in a manner favoring G_s_ binding. These structural features, supported by mutagenesis, indicate that water molecules work in concert with conserved electrostatic and hydrophobic interactions to encode G protein selectivity (Fig 4E–4H, 4J, and 4K). Although internal waters are not clearly resolved in the G_i_ structure, MD simulations suggested rapid reoccupation of the corresponding hydration sites (W_S1_–W_S8_ and W_G1_) and more dynamic water-coordinating interactions than in G_s_, which likely limits direct visualization of these waters. Our findings establish a framework in which modular water networks not only stabilize conserved motifs but also contribute to G protein selectivity.

In summary, our study establishes a modular hydration network in which conserved cavities stabilize activation motifs, while variable cavities such as the EWC shaped by residue 5.58 modulate cavity architecture and hydration. In addition, water molecules at the intracellular interface contribute to G protein selectivity. Together, these results provide mechanistic insight into hydration-mediated regulation of receptor activation and G protein selectivity. More broadly, our study highlights hydration-mediated signaling as a potentially general mechanism of class A GPCR regulation, motivating future high-resolution structures and molecular dynamics simulations to validate the general applicability of this framework across class A GPCRs.

## Materials and methods

### Constructs

For structural determination, wild-type (WT) human GPR174 (UniProt ID: Q9BXC1) was cloned into a modified pFastBac1 vector. An N-terminal hemagglutinin (HA) signal peptide and FLAG tag (DYKDDDDA) were introduced, followed by a thermostabilized BRIL to enhance receptor expression. The C terminus was fused to a LgBiT tag, a TEV protease cleavage site, and tandem maltose-binding protein (MBP) tags. Human dominant-negative (DN) Gα_s_ and DN Gα_i1_ were generated by site-directed mutagenesis as previously described to decrease the nucleotide-binding affinity and increase G protein stability [48]. All three G_s_ subunits, including human DNGα_s_, WT Gβ_1_ fusion to HiBiT, and Gγ_2_, were cloned separately into the pFastBac vector. Similarly, the three G_i_ subunits, including human DNGα_i_, WT Gβ_1_ fusion to HiBiT, and Gγ_2_, were cloned into the pFastBac vector separately [27].

### Expression and purification of Nb35 and scFv16

Nanobody-35 (Nb35) with a C-terminal 6× His tag was expressed in the periplasm of *Escherichia coli* BL21, as described previously [37]. scFv16 containing a C-terminal 6× His tag was constructed and expressed using the Bac-to-Bac system, as described previously [49]. Briefly, the overexpressed Nb35 and scFv16 protein were purified by Ni-NTA resin and eluted by using 500 mM imidazole, respectively. The eluted proteins were then cleaved by TEV protease and further purified by gel filtration chromatography using a Superdex 200 Increase 10/300 GL column. The purified Nb35 and scFv16 fractions were concentrated to approximately 5 mg/mL and stored at −80 °C until use.

### Expression and purification of LysoPS-GPR174-G protein complex

*Spodoptera frugiperda* (Sf9) cells were cultured in ESF921 medium at 27 °C with shaking at 120 rpm. HEK293T cells were maintained in a humidified incubator at 37 °C with 5% CO_2_ in DMEM (VWR) supplemented with 10% fetal bovine serum (FBS, VWR), 100 I.U./mL penicillin, and 100 μg/mL streptomycin (Invitrogen). GPR174 (1–333)-LgBiT, DNGα_s_ or DNGα_i1_, Gβ_1_-HiBiT fusion, and Gγ_2_ were co-expressed in Sf9 insect cells using the Bac-to-Bac baculovirus expression system (Thermo Fisher). The cells were cultured and grown to a density of 2.4 × 10^6^ cells mL^−1^and infected with four separate baculoviruses (GPR174 (1-333)-LgBiT fusion, DNGα_s_ or DNGα_i1_, Gβ_1_-HiBiT fusion, and Gγ_2_) at a ratio of 1:1:1:1. After culturing for 48 h at 27 °C, the cells were harvested by centrifugation and the cell pellets were stored at −80 °C. To purify the GPR174–G_s_ and GPR174–G_i_ complexes, cell pellets were resuspended in buffer containing 20 mM HEPES (pH 7.5), 150 mM NaCl, and 10 mM MgCl_2_, supplemented with protease inhibitor cocktail tablets (Roche). The suspension was homogenized 60 times using a Dounce homogenizer to disrupt cell membranes. Complex formation was initiated by adding 10 μM lysophosphatidylserine (LysoPS) and 25 mU/mL apyrase (Sigma), followed by solubilization with 0.5% (w/v) lauryl maltose neopentyl glycol (LMNG, Anatrace) and 0.1% (w/v) cholesterol hemisuccinate (CHS, Anatrace) for 2 h at 4 °C. The lysate was clarified by centrifugation at 17,000 rpm for 30 min, and the supernatant was incubated with amylose resin for 1 h. After washing with buffer containing 20 mM HEPES (pH 7.5), 150 mM NaCl, 10 mM MgCl_2_, 10 μM LysoPS, 0.01% LMNG, and 0.002% CHS, the complexes were eluted with the same buffer supplemented with 10 mM maltose. TEV protease was added to cleave the MBP tags. The eluted complex was then concentrated and further purified by size-exclusion chromatography on a Superose 6 Increase 10/300 GL column (GE Healthcare) pre-equilibrated with buffer containing 20 mM HEPES (pH 7.5), 150 mM NaCl, 10 mM MgCl_2_, 0.00225% LMNG, 0.00075% GDN, 0.0006% CHS, and 10 μM LysoPS. Peak fractions of the GPR174–G_s_ or GPR174–G_i_ complexes were collected and concentrated to 10 mg/mL using a 100-kDa cutoff Amicon Ultra Centrifugal Filter (Millipore) for cryo-EM analysis.

### Cryo-EM grid preparation and data collection

Holey carbon grids (Quantifoil R1.2/1.3, 300 mesh) were glow-discharged at 25 mA for 1 min using a PELCO easiGlow system. Purified GPR174–G_s_ or GPR174–G_i_ complexes were applied to the glow-discharged grids. Excess sample was blotted for 3.5 s with a blot force of 8 using Ted Pella filter paper at 4 °C and 100% humidity, and the grids were then vitrified in liquid ethane using a Vitrobot Mark IV (Thermo Fisher). Cryo-EM data were collected on a Titan Krios microscope operated at 300 kV, located at the Core Facilities of Zhejiang University Medical Center and Liangzhu Laboratory. For the GPR174–G_s_ complex, data were acquired using EPU software in super-resolution mode with a calibrated pixel size of 0.74 Å and a defocus range of −0.6 to −1.2 μm. Each movie consisted of 30 frames with a total exposure dose of 80 e⁻/Å^2^. For the GPR174–G_i_ complex, micrographs were acquired under similar conditions with a pixel size of 0.93 Å, a defocus range of −1.0 to −2.0 μm, and 40 frames per movie with a total dose of 52 e⁻/Å^2^. In total, 8,814 and 8,490 movies were collected for the GPR174–G_s_ and GPR174–G_i_ complexes, respectively.

### Cryo-EM data processing

Movies were aligned using RELION 4.0 [50], and contrast transfer function (CTF) parameters were estimated with Gctf v1.18 [51]. Subsequent data processing was performed using RELION 4.0 and CryoSPARC v3.3.2 [52]. For the GPR174-G_s_ complex, micrographs were initially processed using RELION 4.0 and CryoSPARC v4.0.3. Template-based particle picking in RELION yielded 4,203,634 particle projections. These were imported into CryoSPARC and subjected to multiple rounds of 2D classification. Selected high-quality particles were used to generate ab initio models, followed by several rounds of heterogeneous refinement in CryoSPARC. A subset of particles was re-extracted and further classified by two additional rounds of 3D classification in RELION to remove poorly defined particles. The resulting 170,885 particles were refined using 3D refinement, CTF refinement, and Bayesian polishing in RELION, yielding a final map with a reported global resolution of 2.0 Å. For the GPR174-G_i_ complex, 3,495,640 particles were initially selected. After similar processing steps, particles were re-extracted into RELION for 3D classification. A final subset of 553,019 particles was used for 3D refinement, CTF refinement, and Bayesian polishing, resulting in a cryo-EM map with a global resolution of 3.4 Å. These reconstructions were used for subsequent model building and analysis.

### Model building and structure refinement

Initial models were generated based on the previously determined structure of LysoPS-bound GPR174 (PDB ID: 7XV3) and AlphaFold3 predictions [53]. The models were manually fitted into the density maps using UCSF Chimera, followed by flexible fitting with Rosetta. Manual model rebuilding was performed in Coot, and real-space refinement was carried out in Phenix. Final refinement statistics were assessed using the Comprehensive Validation (cryo-EM) module in Phenix [54–56]. Model statistics are summarized in S3 Table. Structural figures were prepared using UCSF ChimeraX and PyMOL [57,58].

### Molecular dynamics simulation

#### System preparation.

The cryo-EM structures of the GPR174–G_s_, GPR174–G_i_ complexes, and the P2Y_1_R–G_s_ complex (PDB ID: 7XXH) were used as templates for MD simulations, with missing regions modeled by homology to obtain complete systems [38,59]. Two GPR174 systems were prepared: one retaining the hydration-associated waters observed in the cryo-EM structure, including five ligand-stabilizing waters (W_L1_–W_L5_) and nine waters in the hydration-mediated transmission network (W_S1_–W_S8_ and W_G1_), and the other with these waters removed (GPR174–G_s_–rWat). For GPR174-G_i_, no internal waters were retained, and water occupancy at the predefined sites was analyzed during MD, analogous to the P2Y_1_R simulations. The protonation states were determined using H++ [60]. All the complexes were embedded into an asymmetric lipid bilayer, which was built by the Membrane Builder module in the CHARMM-GUI server [61]. The outer leaflet of the lipid bilayer was composed of 33.3 mol% POPC, 33.3 mol% PSM, and 33.3 mol% cholesterol, whereas the inner leaflet was comprised of 35 mol% POPC, 25 mol% POPE, 20 mol% POPS, and 20 mol% cholesterol. The total numbers of the bilayer membrane lipids for the GPR174-G_s_-Wat, GPR174-G_s_-rWat, GPR174–G_i_ and P2Y_1_R were 495, 495, 498, and 536, respectively. Subsequently, all the systems were solvated in TIP3P solvent containing 0.15 M Na^+^/Cl^−^ and neutralized with Cl^-^, where the dimensions of the simulation boxes were 12.3 × 12.3 × 16.9 nm^3^, 12.3 × 12.3 × 16.9 nm^3^, 12.3 × 12.3 × 15.9 nm^3^, and 12.8 × 12.8 × 17.0 nm^3^, respectively (S5 Table). The CHARMM36m force field was used to describe the system [62], and all MD simulations were performed using GROMACS-2019.4 [63].

#### Simulation protocol.

After 5,000 steps of steepest-descent energy minimization, systems underwent NVT equilibration at 310 K for 250 ps, followed by cumulative 1.65 ns NPT equilibration at 1 atm using the Berendsen barostat [64]. A harmonic positional restraint of 10 kcal/mol·Å^2^ was applied during pre-equilibration and gradually released. Long-range electrostatics were treated by particle-mesh Ewald [65]. Short-range electrostatics and van der Waals interactions used a 10 Å cutoff. All bonds were constrained with LINCS [66]. For production, three independent 1 μs runs were performed for each system with different initial velocities.

#### Analysis.

Hydration-associated waters W_S1_–W_S8_ and W_G1_ were defined from the cryo-EM structure and monitored throughout the trajectories. Hydrogen-bond lifetimes between these waters and coordinating residues were calculated using a custom Python script. The average lifetime was calculated according to the following equation:


$$t_{avg}\;=\;\frac{\sum_{i=\text{1}}^{N_{\text{H}-\text{bond}}}t_{\text{H}-\text{bond},\;i}}{N_{\text{H}-\text{bond}}}$$

where *t*_H-bond, *i*_ represents the lifetime of each hydrogen bond formation, *N*_H-bond_ means the total number of the bond formations, and *t*_*avg*_ means the average lifetime. The hydration-mediated transmission network for the GPR174–G_s_–rWat system was reformed within ~50 ns and remained stable throughout the simulations. Overall system stability was then assessed by calculating the RMSD of heavy atoms, with transmembrane helix Cα atoms. GPR174-G_i_ and P2Y_1_R-G_s_ simulations were analyzed using the same workflow, and internal water occupancy was quantified. All structural analyses and visualizations were performed using UCSF ChimeraX and PyMOL [57,58].

#### NanoBiT G-protein dissociation assay.

G protein activation was measured using the NanoBiT G-protein dissociation assay (Promega) as previously described [67]. To measure G-protein dissociation, HEK293T cells (ATCC: CRL-1573) were co-transfected with WT or mutant GPR174, Gα_s_-LgBiT or Gα_i_-LgBiT, Gβ_1_, and Gγ_2_-SmBiT plasmids at a ratio of 3:1:1:1. Cell seeding and transfection followed the same method as cAMP accumulation assay. The cells were incubated in 96-well plates for over 24 hours at 37 °C in 5% CO_2_. After being washed twice with D-PBS, cells were incubated with 4 nM coelenterazine-400a (Maokangbio) in HBSS supplemented with 5 mM HEPES pH 7.4 and 0.01% BSA for 30 min. The baseline luminescence was read for 5 cycles by SparkControl (TECAN). Then, serially diluted LysoPS was added to each well to stimulate the cells. Luminescence was recorded for an additional 30 min at 300 ms intervals. The raw data were normalized to the baseline and vehicle controls, and dose–response curves were fitted using GraphPad Prism 8.0. EC_50_ and pEC_50_ ± SEM were calculated using nonlinear regression (curve fit). Data are presented as mean ± SEM from at least three independent experiments performed in triplicate.

#### GloSensor cAMP assay.

Intracellular cAMP levels were measured using a GloSensor cAMP assay (Promega). HEK293T cells (ATCC: CRL-1573) were transiently co-transfected with N-terminal FLAG-tagged human P2Y_1_R (WT or mutants) and the GloSensor-22F cAMP biosensor plasmid (Promega) at a ratio of 3:1. At 6 h post-transfection, cells were seeded into 96-well white plates at a density of 3 × 10^4^ cells per well in 80 μL complete DMEM and incubated for >24 h at 37 °C with 5% CO_2_. On the day of assay, culture medium was removed and cells were equilibrated with 40 μL per well CO_2_-independent medium (pH 8.4) containing 2% (v/v) GloSensor cAMP Reagent (Promega) for 30 min at 37 °C. Baseline luminescence was recorded, and cells were stimulated with serially diluted ADP to initiate signaling. Luminescence was monitored on a microplate reader (Berthold Technologies) for 30 min at room temperature. Raw luminescence traces were normalized to baseline and vehicle controls, and dose–response curves were fitted in GraphPad Prism using nonlinear regression. Data are presented as mean ± SEM from at least three independent experiments performed in triplicate.

#### Detection of surface expression of GPR174 mutants.

Cell surface expression levels of WT GPR174 and its mutants were measured by enzyme-linked immunosorbent assay (ELISA). Constructs encoding WT or mutant GPR174 with an N-terminal FLAG tag were cloned into the pcDNA3.1 vector. Cell seeding and transfection followed the same procedures as described for the cAMP accumulation and NanoBiT assays. After 24 hours of incubation, cells were washed twice with phosphate-buffered saline (PBS) and fixed with 4% formaldehyde. Fixed cells were blocked with 1% (w/v) BSA in PBS for 30 min at room temperature, followed by incubation with monoclonal ANTI-FLAG M2-Peroxidase (HRP) antibody (Sigma) diluted 1:10,000 in PBS containing 1% BSA for another 30 min. Afterward, cells were washed three times with PBS containing 1% BSA and three additional times with PBS to remove unbound antibody. SuperSignal ELISA Femto Maximum Sensitivity Substrate (Thermo Fisher Scientific) was added, and luminescence was measured using a TECAN plate reader at 450 nm absorbance. All data were normalized to WT expression and are presented as means ± SEM from at least three independent experiments.

#### Quantification and statistical analysis.

Cell-based luciferase reporter assays were analyzed using Microsoft Excel, while cAMP accumulation and NanoBiT G-protein dissociation assays were analyzed using GraphPad Prism. For all assays, bars and error bars represent the mean and SEM, respectively. Dots indicate individual data points from at least three independent experiments, each performed in duplicate. For the exact sample sizes and details of statistical analysis, refer to the figure legends of Figs 1B, 2C, 2D, 4E–4H, 4J, 4K, S5, S9, and S10.

## Supporting information

S1 Fig Purification and cryo-EM data processing of the LysoPS-bound GPR174-G_s_ complex, related to Fig 1. **(A)** Size-exclusion chromatography (SEC) profile and SDS-PAGE analysis of the purified GPR174-G_s_ complex. Fractions between two dashed lines in the SEC profile were pooled and concentrated for cryo-EM analysis. Uncropped gel for S1A is provided in S1 Raw Images. **(B)** Representative cryo-EM image micrograph (scale bar, 30 nm) and 2D class averages (scale bar, 5 nm) of the GPR174-G_s_ complex. **(C)** Flow chart of cryo-EM data processing and cryo-EM maps of the GPR174-G_s_ complex, colored according to local resolution. **(D)** Fourier shell correlation (FSC) curve of the final refined GPR174-G_s_ map. **(E)** Cryo-EM density maps and models are shown for all seven-transmembrane helices, LysoPS (18:0), and Gα_s_ α5 helix.
(TIF)

S2 Fig Purification and cryo-EM data processing of the GPR174-G_i_ complex, related to Fig 1. **(A)** Size-exclusion chromatography (SEC) profile and SDS-PAGE analysis of the purified GPR174-G_i_ complex. Fractions between two dashed lines in the SEC profile were pooled and concentrated for cryo-EM analysis. Uncropped gel for S2A is provided in S1 Raw Images. **(B)** Representative cryo-EM image micrograph (scale bar, 30 nm) and 2D class averages (scale bar, 5 nm) of the GPR174-G_i_ complex. **(C)** Flow chart of cryo-EM data processing and cryo-EM maps of the GPR174-G_i_ complex, colored according to local resolution. **(D)** Fourier shell correlation (FSC) curve of the final refined GPR174-G_i_ map. **(E)** Cryo-EM density maps and models are shown for all seven-transmembrane helices and Gα_i_ α5 helix.
(TIF)

S3 Fig Structural and functional analyses of the hydration-mediated signaling network of GPR174, related to Fig 2. **(A)** Structural depiction of the hydration-mediated signaling network in the GPR174-G_s_ complex. **(B)** Detailed interactions between LysoPS (purple) and residues within the hydrophobic valley of the orthosteric binding pocket. Hydrophobic residues are shown in pale green sticks; hydrogen bonds are shown as black dashed lines. **(C–G)** Enlarged views of the water molecules focused on the hydration-mediated signaling network in GPR174. Hydrogen bonds forming water-mediated interactions are shown as blue dashed lines, and the residues involved in these interactions are shown with pale green sticks.
(TIF)

S4 Fig Hydrogen-bond stability and reformation of the internal hydration network in the GPR174-G_s_ complex, related to Fig 2. **(A)** Hydrogen-bond lifetimes (ns) between internal waters and surrounding residues in GPR174, comparing cryo-EM-observed water molecules (cryo-EM-water network, orange) and MD-derived water molecules (MD-water network, blue). Quantitative data are listed in Supplementary Table S6. **(B)** Line plot shows the number of internal water sites (W_S1_–W_S8_, and W_G1_) that are simultaneously occupied over time in three independent 1-μs MD trajectories. These water sites were defined based on the cryo-EM structure of the GPR174-G_s_ complex. The dashed horizontal line (*N*_Wat_ ≥ 9) indicates the threshold for hydration network reformation. The orange vertical line marks the ~50-ns time point after which the number of recovered waters stabilizes, indicating rapid and reproducible rehydration. **(C** and **D)** Structural stability of water-coordinating residues was evaluated based on RMSD of the GPR174-G_s_ cryo-EM (C) and GPR174-G_s_ wat-RM (D) systems over the course of the MD simulation. **(E)** Root-mean-square deviation (RMSD) of key residues coordinating either cryo-EM observed waters (cryo-EM, orange) or MD-derived waters (wat-RM, blue), quantifying residue-level stability within the hydration-mediated signaling network.
(TIF)

S5 Fig Hydration-network dynamics and functional validation in the GPR174-G_i_ complex, related to Fig 2. **(A)** Hydrogen-bond lifetimes (ns) between internal waters and surrounding residues in GPR174, comparing cryo-EM-observed water molecules in the GPR174-G_s_ complex (green) with MD-derived water molecules in the GPR174-G_i_ complex (teal). Quantitative data are listed in S7 Table. **(B)** Structural stability of three 1 µs replicate simulations was evaluated based on the RMSD of the GPR174-G_i_ complex over the simulation time. **(C)** Line plot showing the number of internal water sites (W_S1_–W_S8_ and W_G1_) that are simultaneously occupied over time in three independent 1 µs MD trajectories. These sites were defined based on the cryo-EM structure of the GPR174-G_s_ complex. **(D)** RMSD of key residues coordinating internal waters, comparing cryo-EM-observed waters in the GPR174-G_s_ complex (green) with MD-derived waters in the GPR174-G_i_ complex (teal), quantifying residue-level stability within the hydration-mediated signaling network. **(E** and **F)** Dose-response curves of LysoPS (18:0)-induced G_i_ signaling for the indicated hydration-network mutants, displayed in two panels for clarity. Values are mean ± SEM from independent experiments (*n* ≥ 3), each performed in triplicate. Exact n is indicated on the panel. NA, not applicable; ns, *P* > 0.05; *P < 0.05; **P < 0.01; ***P < 0.001; ****P < 0.0001. Statistical significance was assessed using one-way ANOVA followed by Dunnett’s multiple comparisons test versus WT. Statistical analysis results are summarized in S8 Table. The data used to generate graphs in S5E and S5F are available in S1 Data.
(TIF)

S6 Fig Structural comparison and cavity volume analysis of GPCRs with small polar residues at position 5.58, related to Fig 3. Cryo-EM structures of active-state class A GPCRs containing small polar residues at position 5.58, including GPR174 (Q^2.53^/T^5.58^-T^5.61^, this study), P2Y_10_R (H^2.53^/T^5.58^-T^5.61^, PDB ID: 8KGG), GPR55 (L^2.53^/C^5.58^-S^5.61^, PDB ID: 9GE2), PF_2_αR (G^2.53^/I^5.58^-I^5.61^, PDB ID: 8IUK; cavity mainly formed by N^5.56^ and T^5.59^), GPR20 (V^2.53^/S^5.58^-V^5.61^, PDB ID: 8HS3), and H_4_R (V^2.53^/N^5.58^-S^5.61^, PDB ID: 7YFC). Three hydration-associated cavities are highlighted: the Conserved Water Cavity (CWC) near D^2.50^, the Junctional Water Cavity (JWC) near Y^7.53^, and the Extended Water Cavity (EWC) shaped by residues at positions 5.58 and 5.61. Green meshes represent water-accessible volumes identified by parKVFinder and visualized in PyMOL. Key residues forming each cavity are labeled.
(TIF)

S7 Fig Structural comparison and cavity volume analysis of GPCRs with aromatic residues at position 5.58, related to Fig 3. Cryo-EM structures of active-state class A GPCRs containing large hydrophobic or aromatic residues at position 5.58, including GAL_2_R (F^2.53^/Y^5.58^-T^5.61^, PDB ID: 7WQ4), β_2_AR (M^2.53^/Y^5.58^-V^5.61^, PDB ID: 3SN6), P2Y_1_R (Y^2.53^/Y^5.58^-I^5.61^, PDB ID: 7XXH), GPR52 (V^2.53^/T^5.58^-H^5.61^, PDB ID: 6LI3; cavity mainly formed by Y^5.59^ and I^5.62^), FFA_2_R (L^2.53^/Y^5.58^-F^5.61^, PDB ID: 8J24), and GPR119 (I^2.53^/F^5.58^-D^5.61^, PDB ID: 7WCM; cavity mainly formed by F^5.55^ and Y^5.59^). Three hydration-associated cavities are shown: the Conserved Water Cavity (CWC) near D^2.50^, the Junctional Water Cavity (JWC) near Y^7.53^, and the Extended Water Cavity (EWC) shaped by residues at positions 5.58 and 5.61. Orange meshes represent water-accessible volumes identified by parKVFinder and rendered in PyMOL. Key cavity-lining residues are labeled.
(TIF)

S8 Fig Comparative analysis of cavity volumes in relation to residue variation at key cavity-shaping positions, related to Fig 3. **(A)** CWC volumes measured across representative class A GPCRs, arranged from left to right by increasing side chain volume of the residue at position 2.53. **(B)** JWC volumes of class A GPCRs, highlighting that tyrosine is strictly conserved at position 7.53 across all analyzed structures. Cavity volumes were quantified as described in Methods. Receptors analyzed in this figure correspond to those shown in S6 and S7 Figs.
(TIF)

S9 Fig Hydration-mediated transmission network and functional scaffold residues in P2Y_1_R, related to Fig 3. **(A)** MD-derived hydration-mediated transmission network in ADP-bound P2Y_1_R (PDB: 7XXH), showing a water-mediated pathway that extends from conserved core motifs toward the cytoplasmic signaling interface. **(B)** cAMP accumulation assay for P2Y_1_R scaffold-residue mutants, summarized as a relative intrinsic activity (RAi) plot. RAi was calculated as [Span(mutant)/EC_50_(mutant)] divided by [Span(WT)/EC_50_(WT)]. Span and EC_50_ were obtained from averaged concentration-response curves from three independent experiments (*n* ≥ 3). Statistical significance was assessed using one-way ANOVA followed by Dunnett’s multiple comparisons test against the expression-matched WT. ns, *P* > 0.05; *P < 0.05; **P < 0.01; ***P < 0.001; ****P < 0.0001. Exact *P* values (left to right) are: *P* = 0.0310, *P* = 0.0001, *P* = 0.0002, *P* = 0.0086, *P* = 0.0081, P = 0.0015, *P* = 0.0094, and *P* = 0.0028. The data used to generate graphs in S9B is available in S1 Data.
(TIF)

S10 Fig Structural and functional analyses of ligand recognition, activation, and G protein binding modes of GPR174, related to Fig 4. **(A** and **B)** Structural comparison of GPR174-G_s_ and GPR174-G_i_ complexes from extracellular (A) and intracellular (B) views. **(C)** Overlay of LysoPS-binding pockets in GPR174-G_s_ and GPR174-G_i_ complexes, with interacting residues shown as sticks. **(D–G)** Effects of alanine mutations in the LysoPS binding pocket during G_s_ (D and E) and G_i_ (F and G) coupling, measured by NanoBiT dissociation assay. Wild-type curves are shown in dark blue for G_s_ and orange for G_i_; mutants are shown as indicated. **(H)** Comparison of the G protein-binding interface area in GPR174-G_s_ and GPR174-G_i_ complexes, calculated using UCSF Chimera v1.15. **(I–L)** Effects of alanine substitutions at interface-contacting residues on G_s_ (I and J) or G_i_ (K and L) coupling, measured by NanoBiT dissociation assay. Wild-type curves are shown in dark blue for G_s_ and orange for G_i_; mutants are shown as indicated. For all functional curve panels, data are presented as mean ± SEM from at least three independent experiments, each performed in triplicate, and fitted potency and response parameters are reported in S13–S16 Tables. The data used to generate graphs in S10D-S10G and S10I-S10L are available in S1 Data.
(TIF)

S1 Table GPR174-induced dissociation assays of different G proteins, related to Fig 1. (DOCX)

S2 Table Cell surface expression of GPR174 co-expressed with different G proteins, related to Fig 1. (DOCX)

S3 Table Cryo-EM data collection, model refinement and validation statistics, related to Fig 1. (DOCX)

S4 Table GPR174-induced G_s_ dissociation assays of wild-type and mutants at hydration-coordinating residues, related to Fig 2. (DOCX)

S5 Table Details of the all-atomistic molecular dynamics simulations, related to Fig 2. (DOCX)

S6 Table The hydrogen-bond residence time (ns) of the hydration-mediated interactions for the GPR174-G_s_ (Control System) and GPR174-G_s_ (Wat-Rm), related to Fig 2. (DOCX)

S7 Table The hydrogen-bond residence time (ns) of the hydration-mediated interactions for the GPR174-G_s_ and GPR174-G_i_ complexes, related to Fig 2. (DOCX)

S8 Table Potency and efficacy parameters for G_i_ signaling of hydration-network mutants, related to Fig 2. (DOCX)

S9 Table Cell-surface expression levels of GPR174 hydration-network mutants in the G_i_ signaling assay, determined by ELISA, related to Fig 2. (DOCX)

S10 Table Hydration cavity volumes in active-state class A GPCRs, related to Fig 3. (DOCX)

S11 Table P2Y_1_R-induced cAMP accumulation assays of wild-type and mutant P2Y_1_R, related to Fig 3. (DOCX)

S12 Table Cell-surface expression levels of wild-type and mutant P2Y_1_R, determined by ELISA, related to Fig 3. (DOCX)

S13 Table Cell surface expression of wild-type and mutant GPR174 co-expressed with G_s_, related to Figs 2 and 4. (DOCX)

S14 Table GPR174-induced G_s_ dissociation assays of wild-type and mutant GPR174, related to Fig 4. (DOCX)

S15 Table GPR174-induced G_i_ dissociation assays of wild-type and mutant GPR174, related to Fig 4. (DOCX)

S16 Table Cell surface expression of wild-type and mutant GPR174 co-expressed with G_i_, related to Fig 4. (DOCX)

S1 Data Data used for graphs in Figs 1B, 2C, 2D, 4E–4H, 4J, 4K, S5E, S5F, S9B, S10D–S10G, and S10I–S10L. (XLSX)

S1 Raw Images Uncropped Coomassie-stained SDS-PAGE gels used for S1A and S2A Figs. (PDF)

## Abbreviations

CTF: contrast transfer function
cryo-EM: cryo-electron microscopy
CWC: conserved water cavity
DN: dominant-negative
ELISA: enzyme-linked immunosorbent assay
FBS: fetal bovine serum
GPCRs: G protein-coupled receptors
LMNG: lauryl maltose neopentyl glycol
LysoPS: lysophosphatidylserine
MBP: maltose-binding protein
PBS: phosphate-buffered saline
WT: wild-type
