# Supplementary figures and images for: Structured water molecules drive activation and G protein selectivity in the GPR174 receptor
Source: PLoS Biol. 2026 May 7;24(5):e3003447. doi: 10.1371/journal.pbio.3003447 (PMC13152116; doi:10.1371/journal.pbio.3003447)

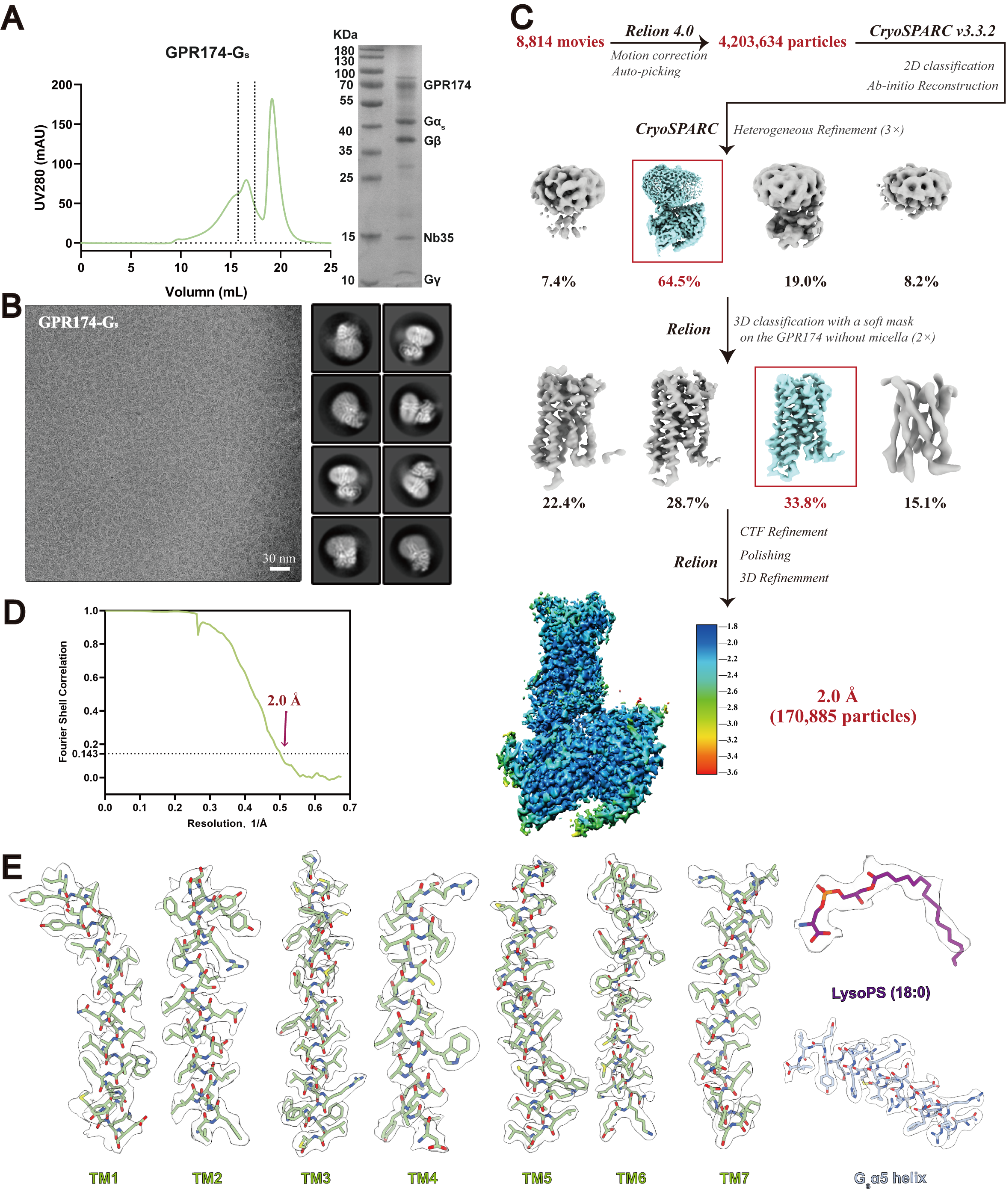

Supplement: S1 Fig — (A) Size-exclusion chromatography (SEC) profile and SDS-PAGE analysis of the purified GPR174-Gs complex. Fractions between two dashed lines in the SEC profile were pooled and concentrated for cryo-EM analysis. Uncropped gel for S1A is provided in S1 Raw Images. (B) Representative cryo-EM image micrograph (scale bar, 30 nm) and 2D class averages (scale bar, 5 nm) of the GPR174-Gs complex. (C) Flow chart of cryo-EM data processing and cryo-EM maps of the GPR174-Gs complex, colored according to local resolution. (D) Fourier shell correlation (FSC) curve of the final refined GPR174-Gs map. (E) Cryo-EM density maps and models are shown for all seven-transmembrane helices, LysoPS (18:0), and Gαs α5 helix. (TIF) [file pbio.3003447.s001.tif]

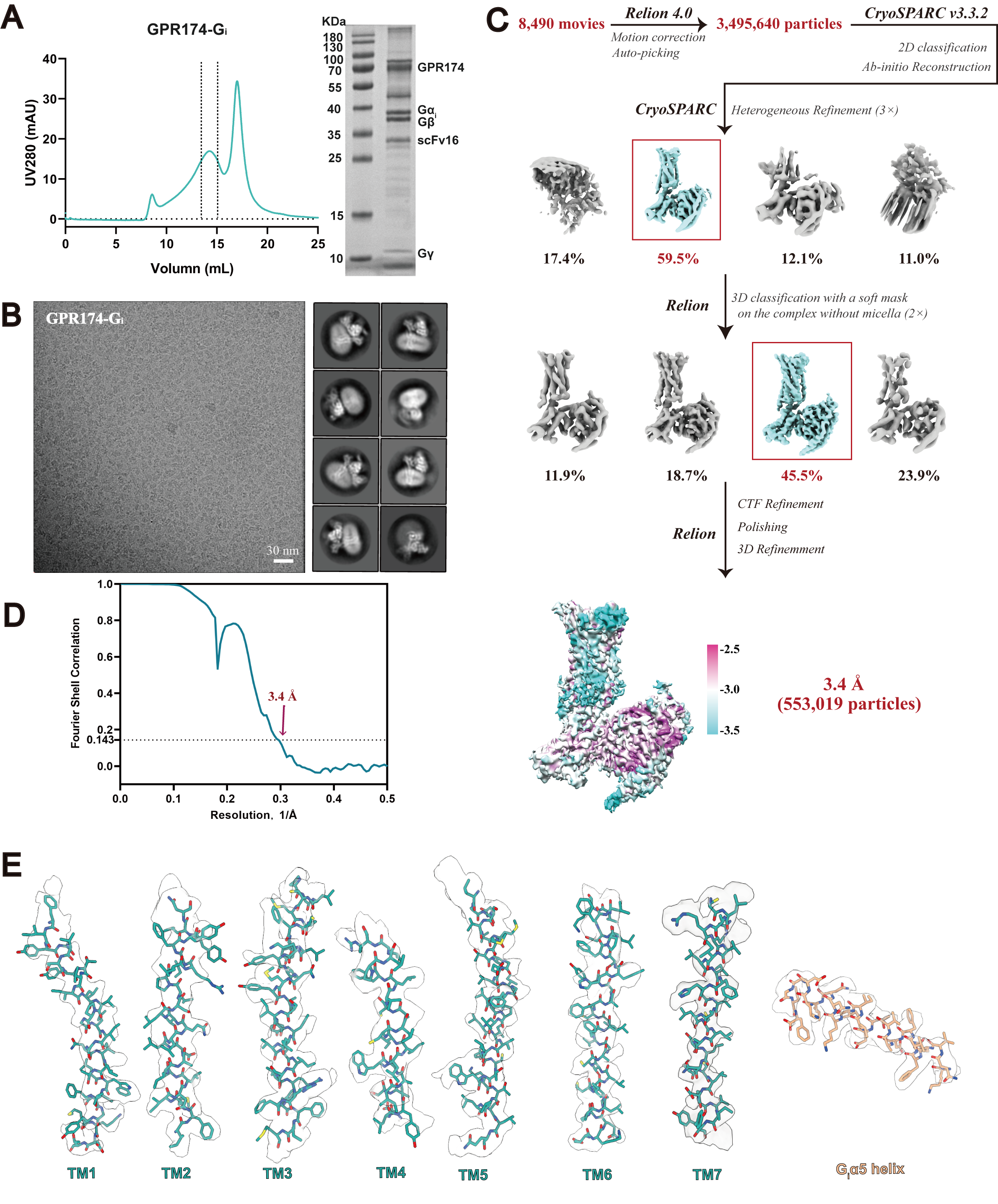

Supplement: S2 Fig — (A) Size-exclusion chromatography (SEC) profile and SDS-PAGE analysis of the purified GPR174-Gi complex. Fractions between two dashed lines in the SEC profile were pooled and concentrated for cryo-EM analysis. Uncropped gel for S2A is provided in S1 Raw Images. (B) Representative cryo-EM image micrograph (scale bar, 30 nm) and 2D class averages (scale bar, 5 nm) of the GPR174-Gi complex. (C) Flow chart of cryo-EM data processing and cryo-EM maps of the GPR174-Gi complex, colored according to local resolution. (D) Fourier shell correlation (FSC) curve of the final refined GPR174-Gi map. (E) Cryo-EM density maps and models are shown for all seven-transmembrane helices and Gαi α5 helix. (TIF) [file pbio.3003447.s002.tif]

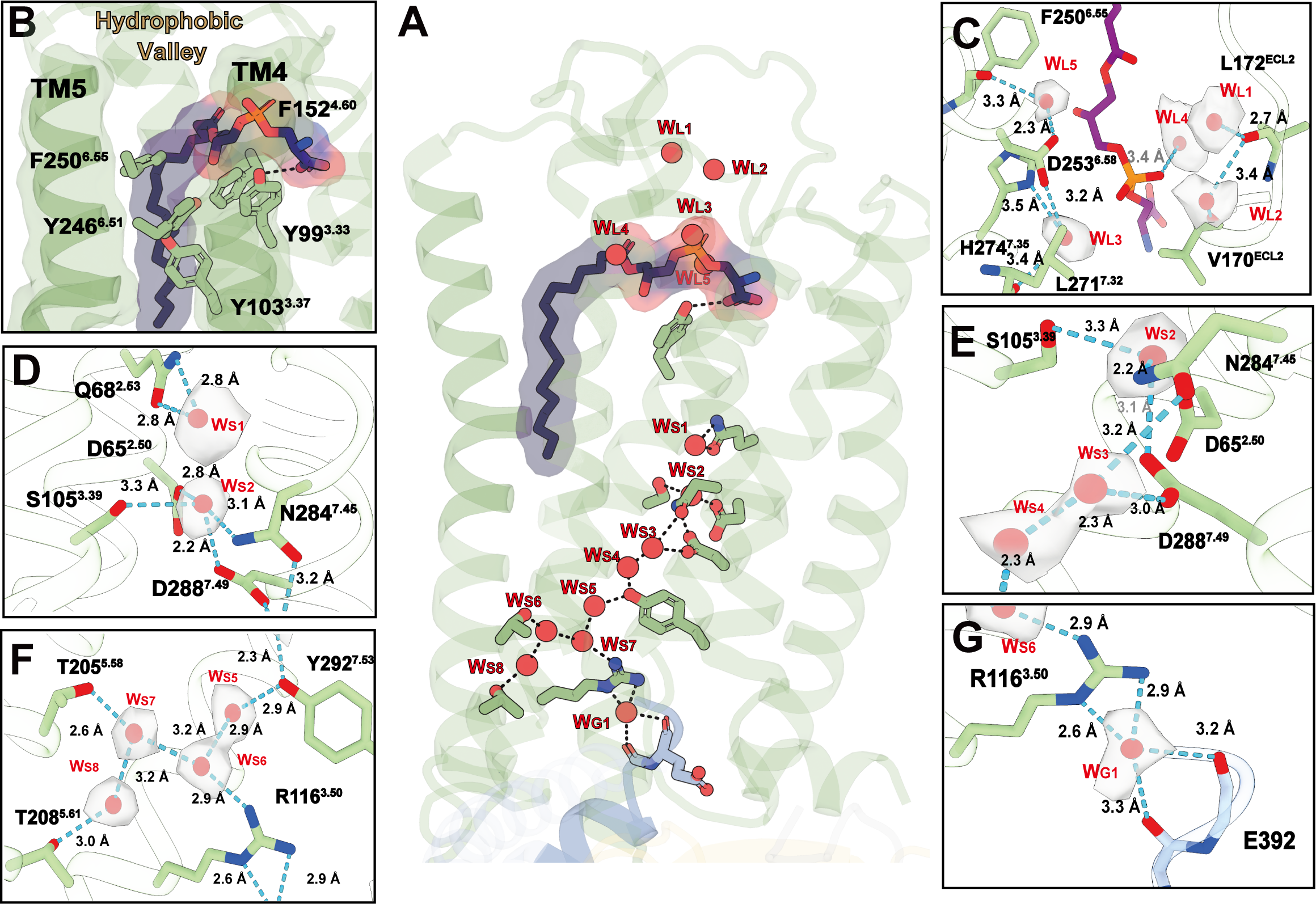

Supplement: S3 Fig — (A) Structural depiction of the hydration-mediated signaling network in the GPR174-Gs complex. (B) Detailed interactions between LysoPS (purple) and residues within the hydrophobic valley of the orthosteric binding pocket. Hydrophobic residues are shown in pale green sticks; hydrogen bonds are shown as black dashed lines. (C–G) Enlarged views of the water molecules focused on the hydration-mediated signaling network in GPR174. Hydrogen bonds forming water-mediated interactions are shown as blue dashed lines, and the residues involved in these interactions are shown with pale green sticks. (TIF) [file pbio.3003447.s003.tif]

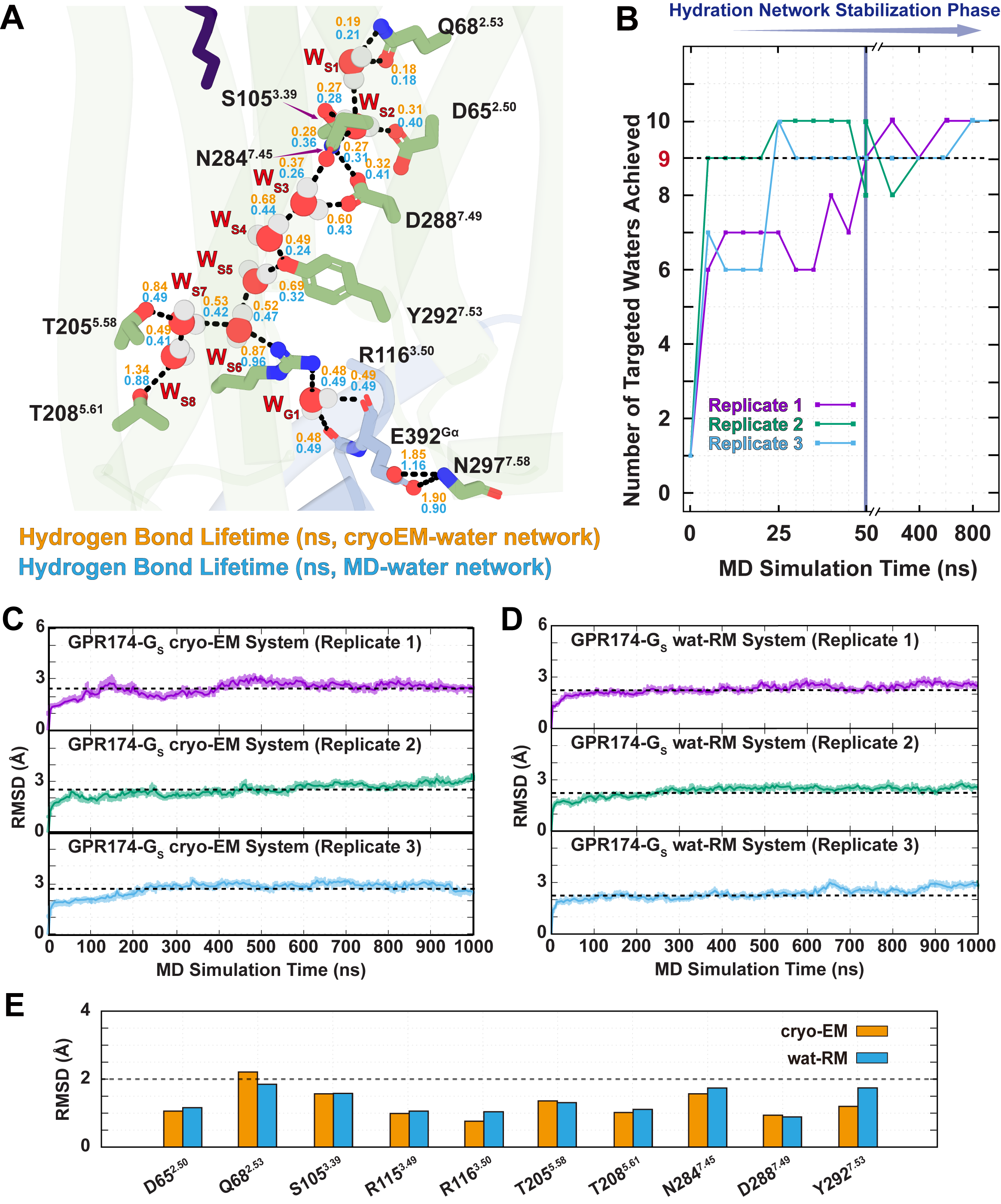

Supplement: S4 Fig — (A) Hydrogen-bond lifetimes (ns) between internal waters and surrounding residues in GPR174, comparing cryo-EM-observed water molecules (cryo-EM-water network, orange) and MD-derived water molecules (MD-water network, blue). Quantitative data are listed in Supplementary Table S6. (B) Line plot shows the number of internal water sites (WS1–WS8, and WG1) that are simultaneously occupied over time in three independent 1-μs MD trajectories. These water sites were defined based on the cryo-EM structure of the GPR174-Gs complex. The dashed horizontal line (NWat ≥ 9) indicates the threshold for hydration network reformation. The orange vertical line marks the ~50-ns time point after which the number of recovered waters stabilizes, indicating rapid and reproducible rehydration. (C and D) Structural stability of water-coordinating residues was evaluated based on RMSD of the GPR174-Gs cryo-EM (C) and GPR174-Gs wat-RM (D) systems over the course of the MD simulation. (E) Root-mean-square deviation (RMSD) of key residues coordinating either cryo-EM observed waters (cryo-EM, orange) or MD-derived waters (wat-RM, blue), quantifying residue-level stability within the hydration-mediated signaling network. (TIF) [file pbio.3003447.s004.tif]

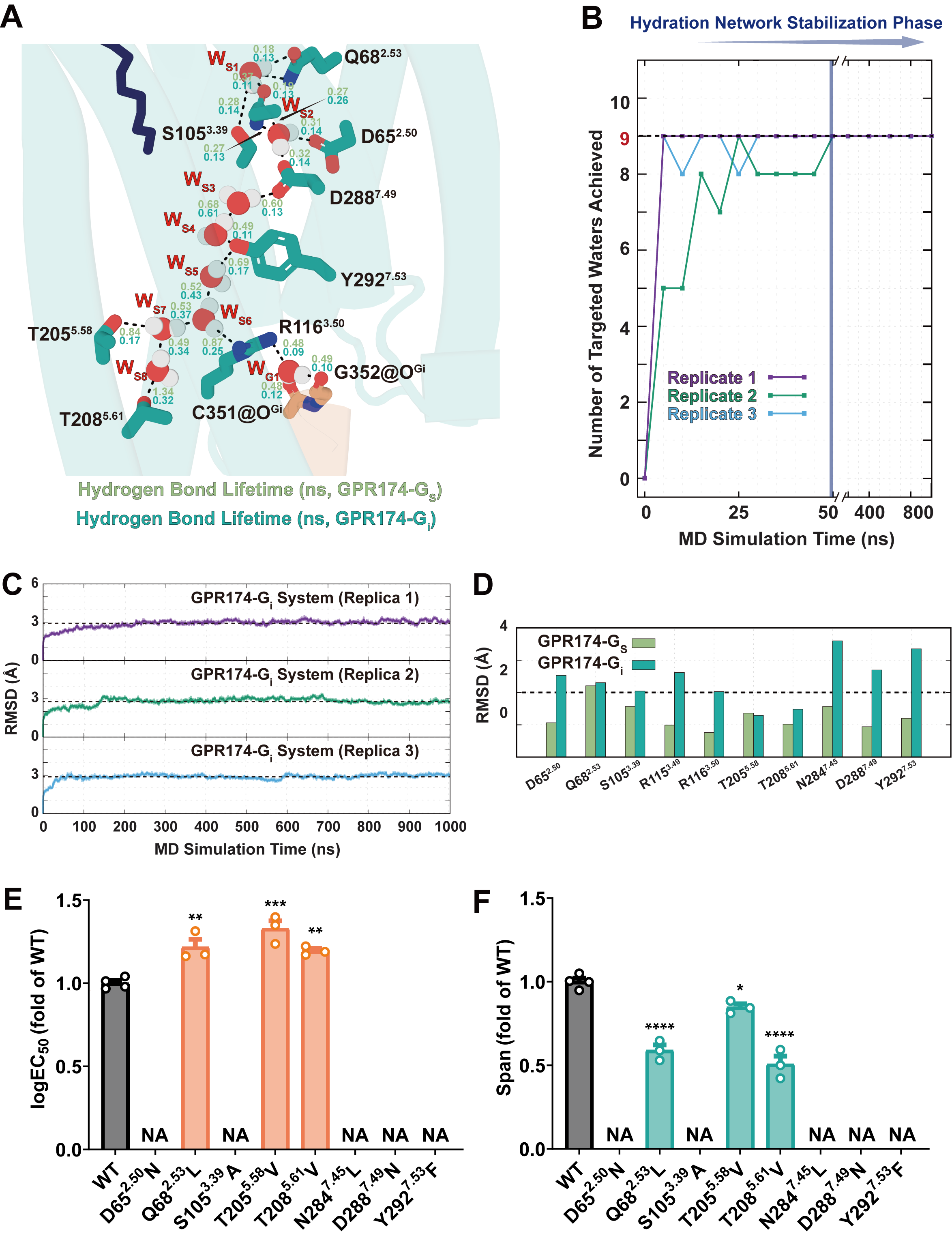

Supplement: S5 Fig — (A) Hydrogen-bond lifetimes (ns) between internal waters and surrounding residues in GPR174, comparing cryo-EM-observed water molecules in the GPR174-Gs complex (green) with MD-derived water molecules in the GPR174-Gi complex (teal). Quantitative data are listed in S7 Table. (B) Structural stability of three 1 µs replicate simulations was evaluated based on the RMSD of the GPR174-Gi complex over the simulation time. (C) Line plot showing the number of internal water sites (WS1–WS8 and WG1) that are simultaneously occupied over time in three independent 1 µs MD trajectories. These sites were defined based on the cryo-EM structure of the GPR174-Gs complex. (D) RMSD of key residues coordinating internal waters, comparing cryo-EM-observed waters in the GPR174-Gs complex (green) with MD-derived waters in the GPR174-Gi complex (teal), quantifying residue-level stability within the hydration-mediated signaling network. (E and F) Dose-response curves of LysoPS (18:0)-induced Gi signaling for the indicated hydration-network mutants, displayed in two panels for clarity. Values are mean ± SEM from independent experiments (n ≥ 3), each performed in triplicate. Exact n is indicated on the panel. NA, not applicable; ns, P > 0.05; *P < 0.05; **P < 0.01; ***P < 0.001; ****P < 0.0001. Statistical significance was assessed using one-way ANOVA followed by Dunnett’s multiple comparisons test versus WT. Statistical analysis results are summarized in S8 Table. The data used to generate graphs in S5E and S5F are available in S1 Data. (TIF) [file pbio.3003447.s005.tif]

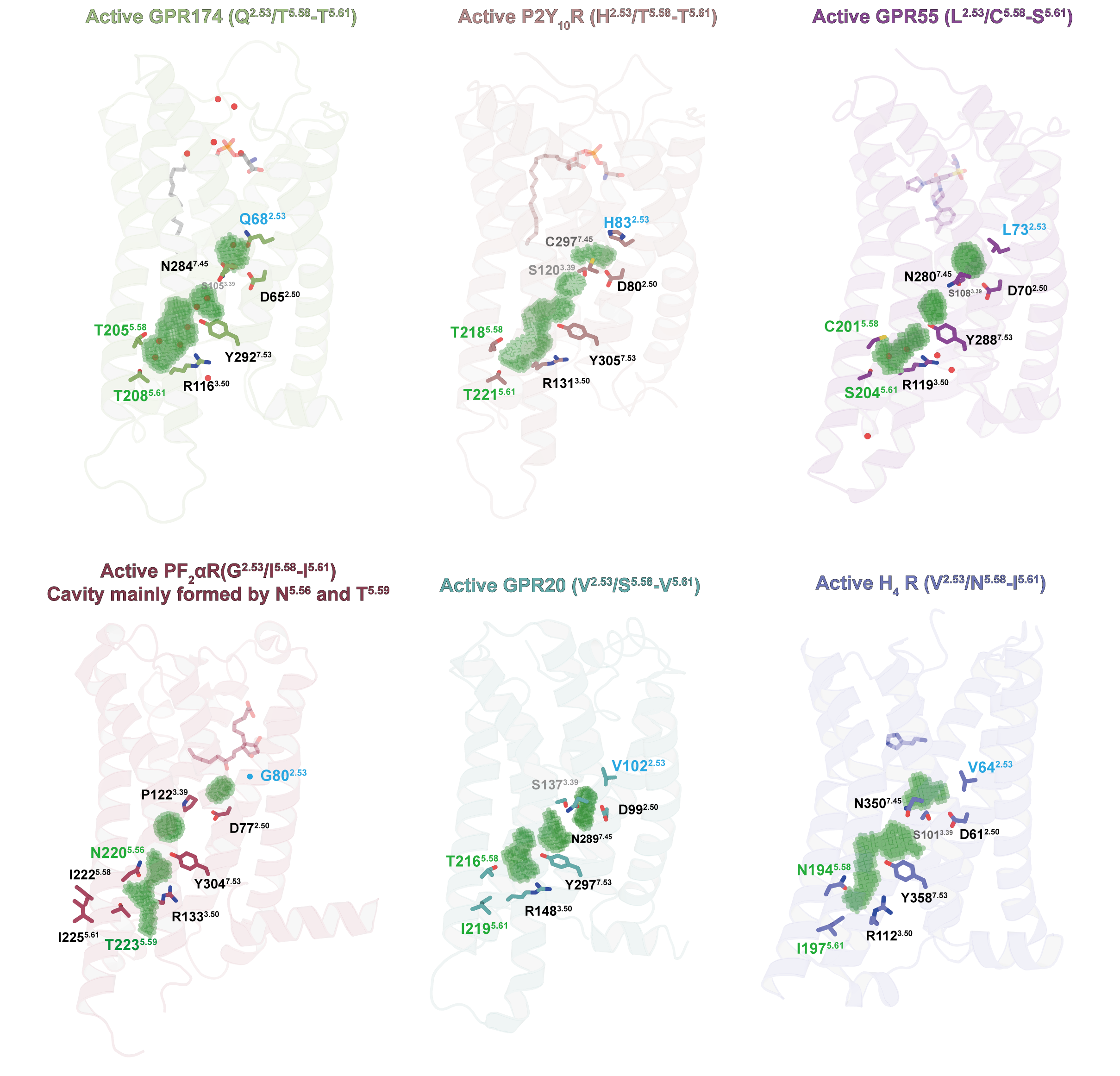

Supplement: S6 Fig — Cryo-EM structures of active-state class A GPCRs containing small polar residues at position 5.58, including GPR174 (Q2.53/T5.58-T5.61, this study), P2Y10R (H2.53/T5.58-T5.61, PDB ID: 8KGG), GPR55 (L2.53/C5.58-S5.61, PDB ID: 9GE2), PF2αR (G2.53/I5.58-I5.61, PDB ID: 8IUK; cavity mainly formed by N5.56 and T5.59), GPR20 (V2.53/S5.58-V5.61, PDB ID: 8HS3), and H4R (V2.53/N5.58-S5.61, PDB ID: 7YFC). Three hydration-associated cavities are highlighted: the Conserved Water Cavity (CWC) near D2.50, the Junctional Water Cavity (JWC) near Y7.53, and the Extended Water Cavity (EWC) shaped by residues at positions 5.58 and 5.61. Green meshes represent water-accessible volumes identified by parKVFinder and visualized in PyMOL. Key residues forming each cavity are labeled. (TIF) [file pbio.3003447.s006.tif]

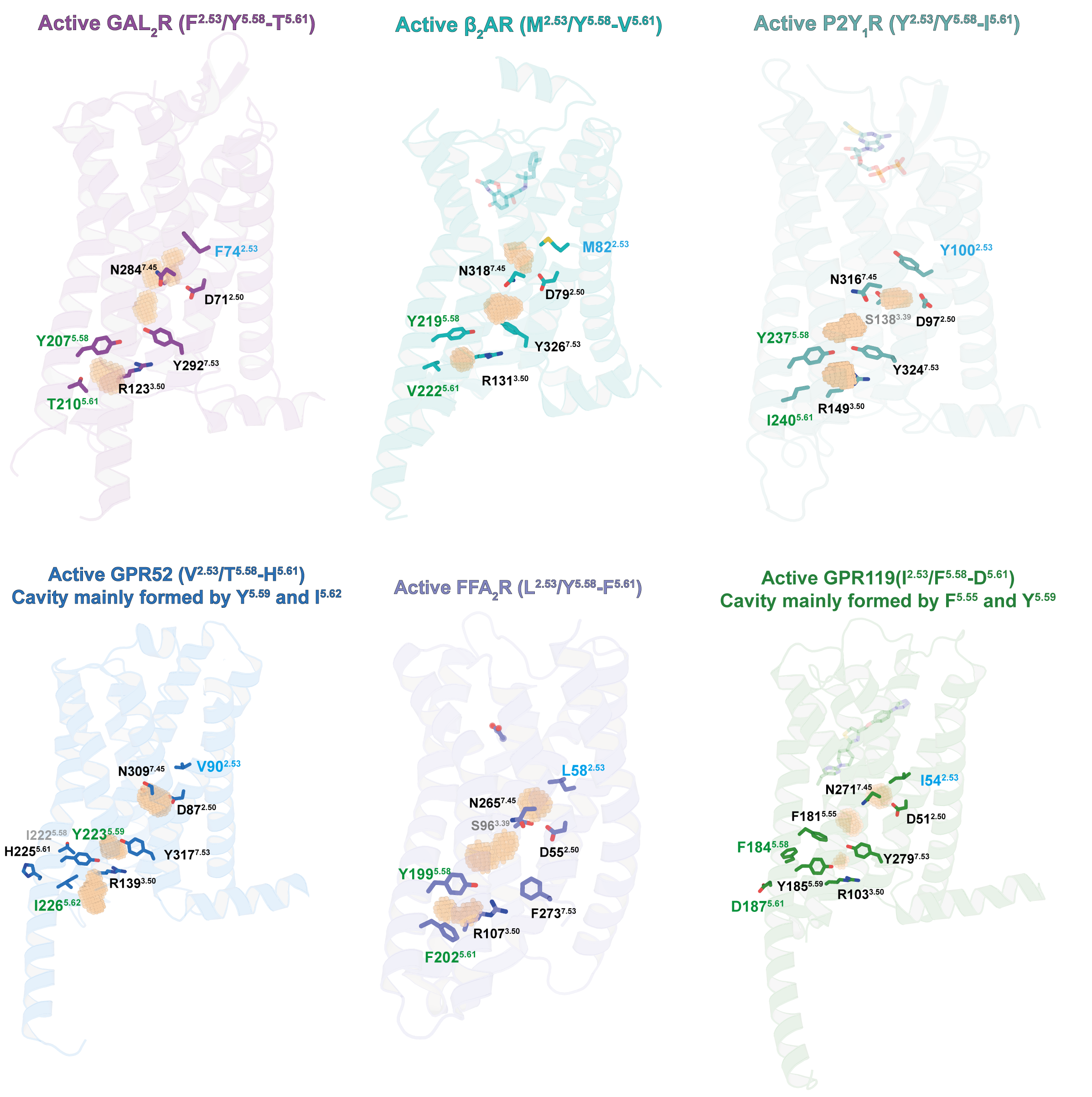

Supplement: S7 Fig — Cryo-EM structures of active-state class A GPCRs containing large hydrophobic or aromatic residues at position 5.58, including GAL2R (F2.53/Y5.58-T5.61, PDB ID: 7WQ4), β2AR (M2.53/Y5.58-V5.61, PDB ID: 3SN6), P2Y1R (Y2.53/Y5.58-I5.61, PDB ID: 7XXH), GPR52 (V2.53/T5.58-H5.61, PDB ID: 6LI3; cavity mainly formed by Y5.59 and I5.62), FFA2R (L2.53/Y5.58-F5.61, PDB ID: 8J24), and GPR119 (I2.53/F5.58-D5.61, PDB ID: 7WCM; cavity mainly formed by F5.55 and Y5.59). Three hydration-associated cavities are shown: the Conserved Water Cavity (CWC) near D2.50, the Junctional Water Cavity (JWC) near Y7.53, and the Extended Water Cavity (EWC) shaped by residues at positions 5.58 and 5.61. Orange meshes represent water-accessible volumes identified by parKVFinder and rendered in PyMOL. Key cavity-lining residues are labeled. (TIF) [file pbio.3003447.s007.tif]

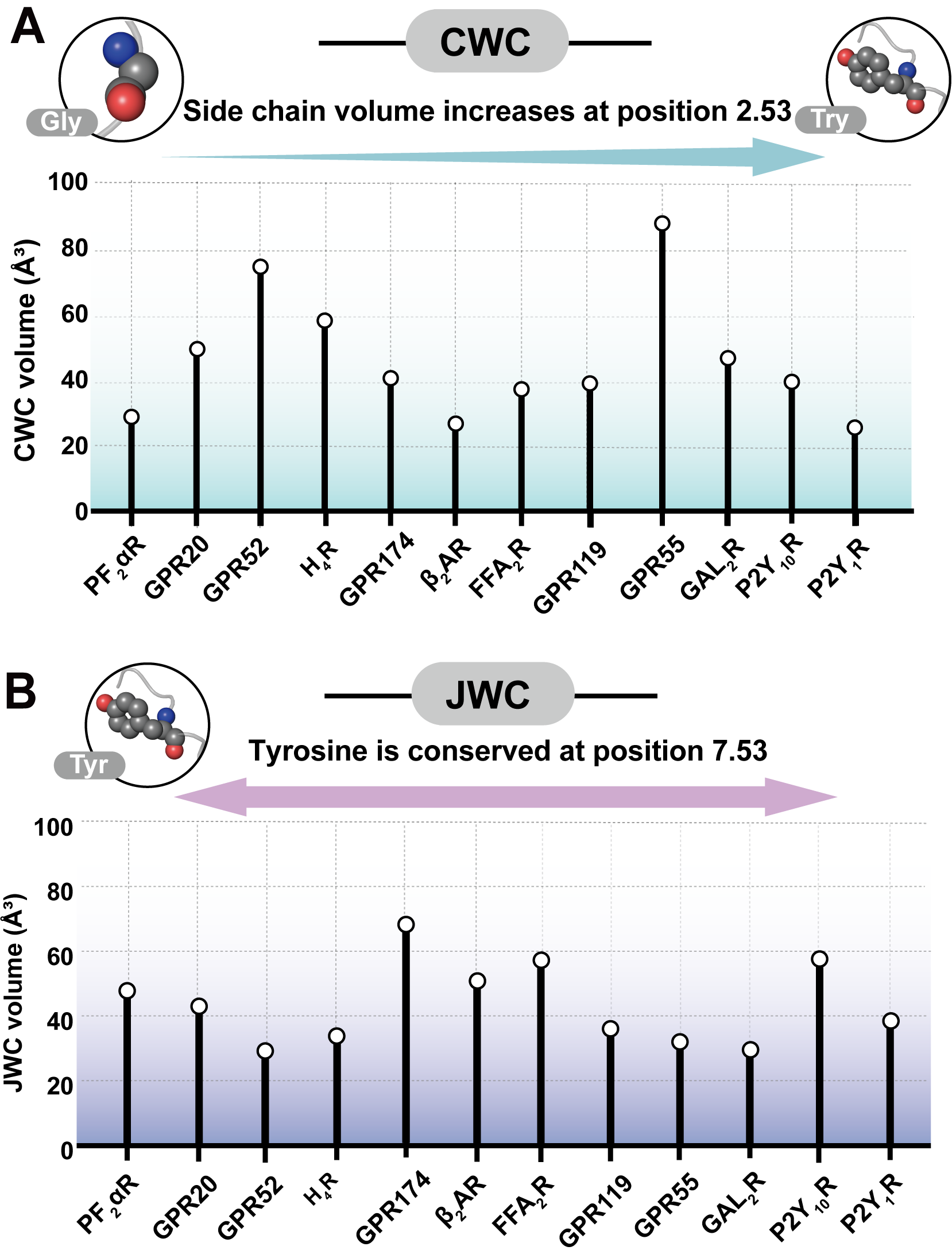

Supplement: S8 Fig — (A) CWC volumes measured across representative class A GPCRs, arranged from left to right by increasing side chain volume of the residue at position 2.53. (B) JWC volumes of class A GPCRs, highlighting that tyrosine is strictly conserved at position 7.53 across all analyzed structures. Cavity volumes were quantified as described in Methods. Receptors analyzed in this figure correspond to those shown in S6 and S7 Figs. (TIF) [file pbio.3003447.s008.tif]

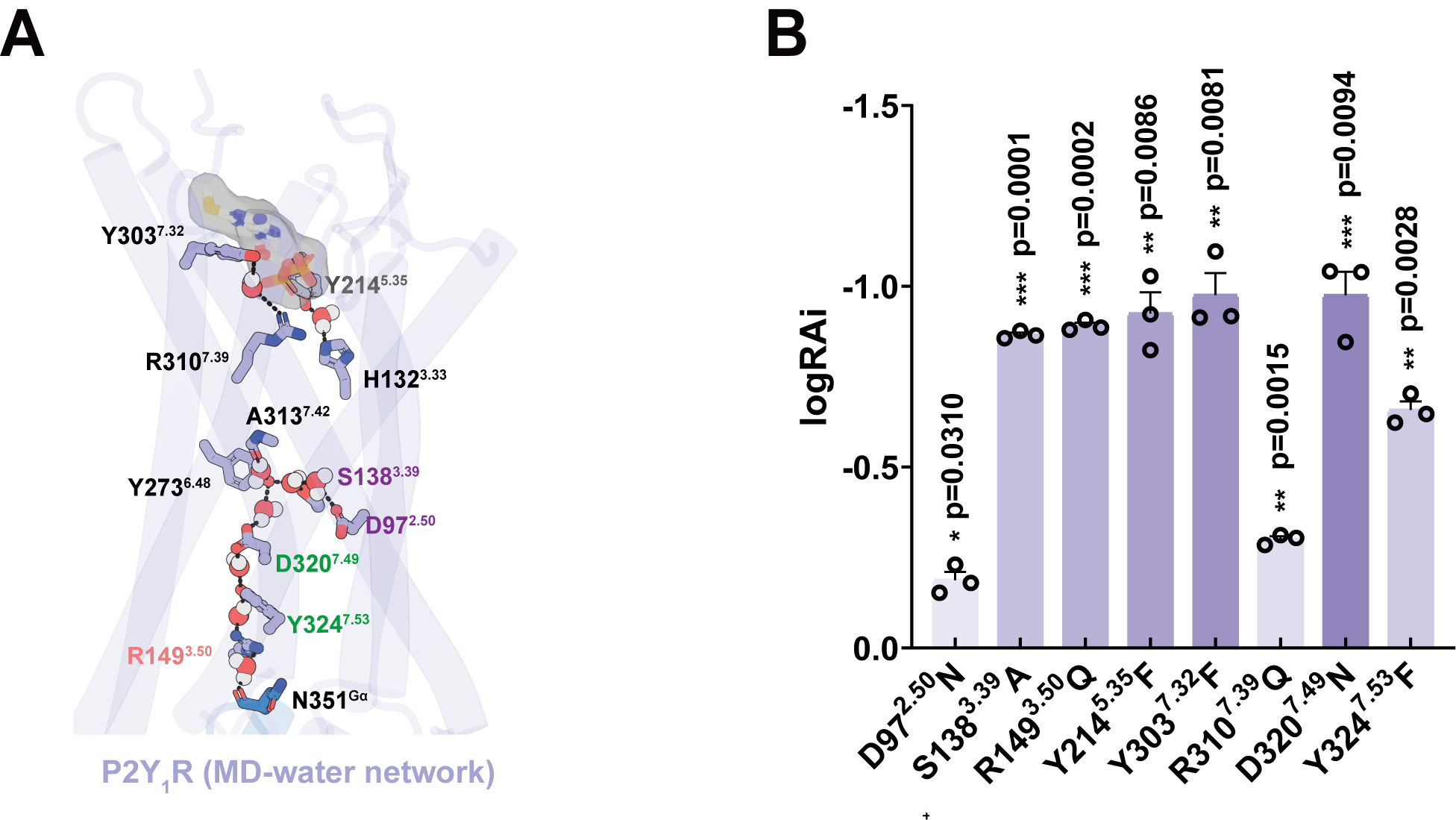

Supplement: S9 Fig — (A) MD-derived hydration-mediated transmission network in ADP-bound P2Y1R (PDB: 7XXH), showing a water-mediated pathway that extends from conserved core motifs toward the cytoplasmic signaling interface. (B) cAMP accumulation assay for P2Y1R scaffold-residue mutants, summarized as a relative intrinsic activity (RAi) plot. RAi was calculated as [Span(mutant)/EC50(mutant)] divided by [Span(WT)/EC50(WT)]. Span and EC50 were obtained from averaged concentration-response curves from three independent experiments (n ≥ 3). Statistical significance was assessed using one-way ANOVA followed by Dunnett’s multiple comparisons test against the expression-matched WT. ns, P > 0.05; *P < 0.05; **P < 0.01; ***P < 0.001; ****P < 0.0001. Exact P values (left to right) are: P = 0.0310, P = 0.0001, P = 0.0002, P = 0.0086, P = 0.0081, P = 0.0015, P = 0.0094, and P = 0.0028. The data used to generate graphs in S9B is available in S1 Data. (TIF) [file pbio.3003447.s009.tif]

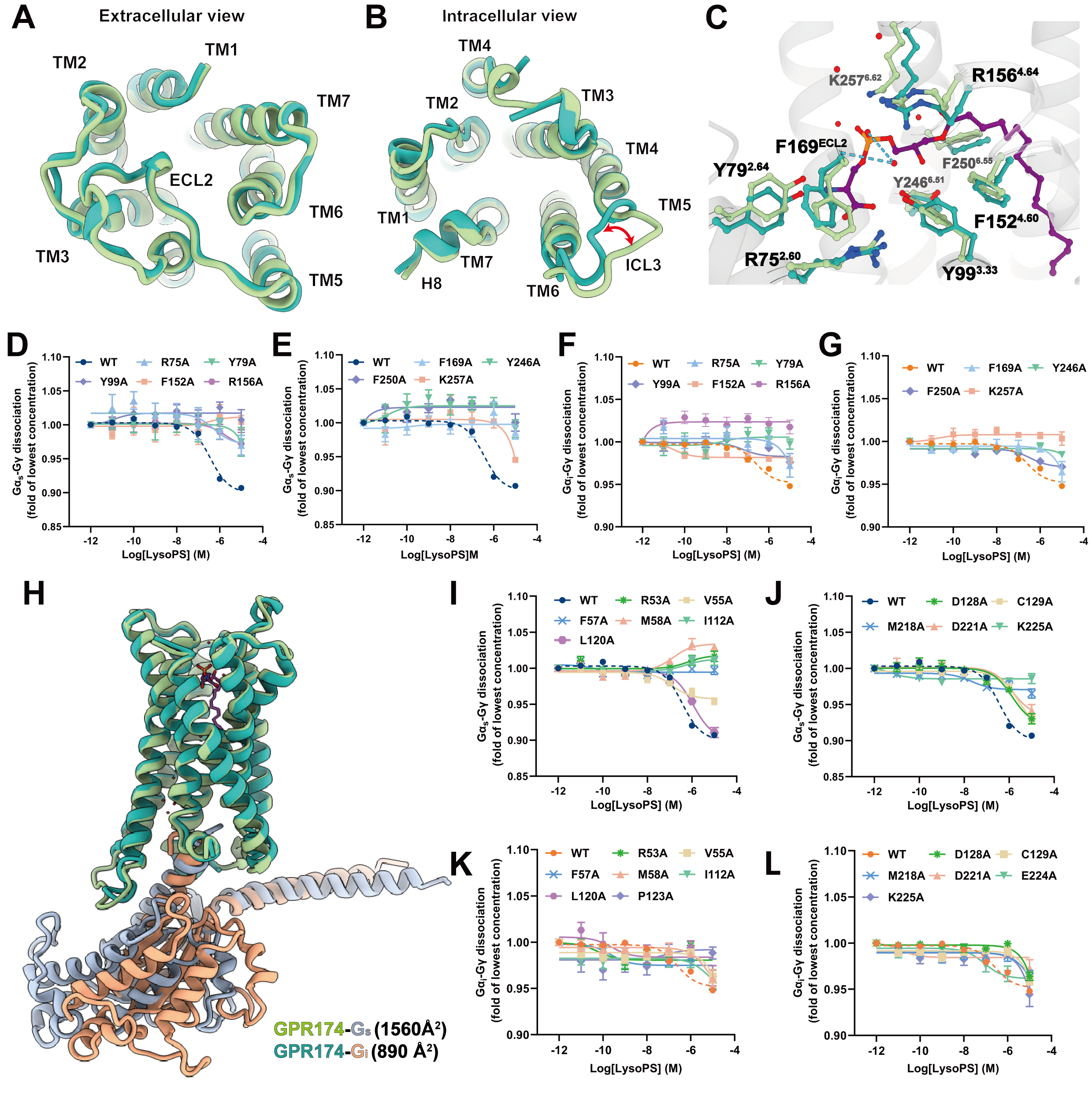

Supplement: S10 Fig — (A and B) Structural comparison of GPR174-Gs and GPR174-Gi complexes from extracellular (A) and intracellular (B) views. (C) Overlay of LysoPS-binding pockets in GPR174-Gs and GPR174-Gi complexes, with interacting residues shown as sticks. (D–G) Effects of alanine mutations in the LysoPS binding pocket during Gs (D and E) and Gi (F and G) coupling, measured by NanoBiT dissociation assay. Wild-type curves are shown in dark blue for Gs and orange for Gi; mutants are shown as indicated. (H) Comparison of the G protein-binding interface area in GPR174-Gs and GPR174-Gi complexes, calculated using UCSF Chimera v1.15. (I–L) Effects of alanine substitutions at interface-contacting residues on Gs (I and J) or Gi (K and L) coupling, measured by NanoBiT dissociation assay. Wild-type curves are shown in dark blue for Gs and orange for Gi; mutants are shown as indicated. For all functional curve panels, data are presented as mean ± SEM from at least three independent experiments, each performed in triplicate, and fitted potency and response parameters are reported in S13–S16 Tables. The data used to generate graphs in S10D-S10G and S10I-S10L are available in S1 Data. (TIF) [file pbio.3003447.s010.tif]
