# Supplementary material for: Structured water molecules drive activation and G protein selectivity in the GPR174 receptor
Source: PLoS Biol. 2026 May 7;24(5):e3003447. doi: 10.1371/journal.pbio.3003447 (PMC13152116; doi:10.1371/journal.pbio.3003447)
Supplement: S1 Table — (DOCX) [file pbio.3003447.s011.docx]

**S1 Table. GPR174-induced dissociation assays of different G proteins, related to Figure 1.**

| G proteins | pEC_50_ ± SEM | Span ± SEM | Sample size |
| --- | --- | --- | --- |
| G_s_ | 6.496±0.141 | -0.100±0.004 | 5 |
| G_i_ | 6.580±0.307 | -0.048±0.009 | 4 |
| G_q_ | NA | NA | 3 |
| G_13_ | 7.472±0.738 | -0.035±0.006 | 3 |

Data were analyzed using a three-parameter logistic equation to determine pEC_50_ and Span. All data are shown as mean ± SEM from at least three independent experiments, each performed in triplicate. Values in S1 Data are shown as the mean of triplicates for each independent experiment. NA, not applicable.
