# Supplementary material for: Structured water molecules drive activation and G protein selectivity in the GPR174 receptor
Source: PLoS Biol. 2026 May 7;24(5):e3003447. doi: 10.1371/journal.pbio.3003447 (PMC13152116; doi:10.1371/journal.pbio.3003447)
Supplement: S2 Table — (DOCX) [file pbio.3003447.s012.docx]

**S2 Table. Cell surface expression of GPR174 co-expressed with different G proteins,** **related to Figure 1.**

| G proteins | Expression ± SEM (% G_s_) | Sample size |
| --- | --- | --- |
| G_s_ | 100±3 | 6 |
| G_i_ | 101±5 | 3 |
| G_q_ | 92±6 | 3 |
| G_13_ | 92±9 | 3 |

Data are shown as mean ± SEM from at least three independent experiments, each performed in triplicate.
