# Supplementary material for: Structured water molecules drive activation and G protein selectivity in the GPR174 receptor
Source: PLoS Biol. 2026 May 7;24(5):e3003447. doi: 10.1371/journal.pbio.3003447 (PMC13152116; doi:10.1371/journal.pbio.3003447)
Supplement: S3 Table — (DOCX) [file pbio.3003447.s013.docx]

**S3 Table. Cryo-EM data collection, model refinement and validation statistics, related to Figure 1.**

|  | GPR174-G_s_ complex | GPR174-G_i_ complex |
| --- | --- | --- |
| Data collection and processing |  |  |
| Magnification | 150,540 | 150,540 |
| Voltage (kV) | 300 | 300 |
| Electron exposure (e^–^/Å^2^) | 80 | 52 |
| Defocus range (μm) | -0.6 ~ -1.2 | -1.0 ~ -2.0 |
| Pixel size (Å) | 0.74 | 0.93 |
| Symmetry imposed | C1 | C1 |
| Initial particle projections (no.) | 4,203,634 | 3,495,640 |
| Final particle projections (no.) | 170,885 | 553,019 |
| Map resolution (Å) | 2.0 | 3.4 |
| FSC threshold | 0.143 | 0.143 |
| Map resolution range (Å) | 1.9-3.0 | 2.8-4.0 |
|  |  |  |
| Refinement |  |  |
| Initial model used | AlphaFold3 and 7XV3 | AlphaFold3 and 7XV3 |
| Model resolution (Å)  FSC threshold | 2.0  0.5 | 3.4  0.5 |
| Model resolution range (Å) | 1.9-3.0 | 2.8-4.0 |
| Map sharpening method | DeepEMhancer | DeepEMhancer |
| Model composition  Non-hydrogen atoms  Protein residues | 8,333  1,044 | 7,096  901 |
| *B* factors (Å^2^)  Protein  Ligand | 77.67  96.55 | 88.25  - |
| R.m.s. deviations  Bond lengths (Å)  Bond angles (°) | 0.003  0.617 | 0.006  0.734 |
| Validation  MolProbity score  Clashscore  Rotamer outliers (%) | 1.49  4.97  0.00 | 1.82  10.75  0.11 |
| Ramachandran plot  Favored (%)  Allowed (%)  Disallowed (%) | 96.50  3.50  0.00 | 96.63  3.26  0.00 |
