# Supplementary material for: Structured water molecules drive activation and G protein selectivity in the GPR174 receptor
Source: PLoS Biol. 2026 May 7;24(5):e3003447. doi: 10.1371/journal.pbio.3003447 (PMC13152116; doi:10.1371/journal.pbio.3003447)
Supplement: S4 Table — (DOCX) [file pbio.3003447.s014.docx]

**S4 Table. GPR174-induced** **G**_s_ **dissociation assays of wild-type and mutants at hydration-coordinating residues, related to Figure 2.**

| Mutation | pEC_50_ ± SEM^a^ | Span ± SEM (% WT)^a^ | Sample size |
| --- | --- | --- | --- |
| WT | 6.496±0.141 | 100.000±3.817 | 5 |
| D65^2.50^N | NA | NA | 3 |
| Q68^2.53^L | 8.704±0.664^***^ | 33.726±5.946^****^ | 3 |
| S105^3.39^A | 6.775±0.077^ns^ | 56.354±3.837^****^ | 3 |
| R116^3.50^Q | NA | NA | 3 |
| T205^5.58^V | 7.290±0.577^ns^ | 65.441±6.216^****^ | 3 |
| T208^5.61^V | 7.687±0.170^ns^ | 53.016±1.126^****^ | 3 |
| N284^7.45^L | 6.020±0.286^ns^ | 79.301±5.979^*^ | 3 |
| D288^7.49^N | 7.273±0.336^ns^ | 54.259±3.746^****^ | 3 |
| Y292^7.53^F | 8.270±0.040^**^ | 43.263±1.718^****^ | 3 |

Data were analyzed using a three-parameter logistic equation to determine pEC_50_ and Span. Span values were normalized to the WT, which was set to 100%. All data are presented as mean ± SEM from at least three independent experiments, each performed in triplicate. Values in S1 Data are shown as the mean of triplicates for each independent experiment. NA, not applicable. ns, not significant; *P < 0.05; **P < 0.01; ***P < 0.001; ****P < 0.0001 by one-way ANOVA followed by Dunnett’s multiple comparisons test, compared with the response of the WT.
