# Supplementary material for: Structured water molecules drive activation and G protein selectivity in the GPR174 receptor
Source: PLoS Biol. 2026 May 7;24(5):e3003447. doi: 10.1371/journal.pbio.3003447 (PMC13152116; doi:10.1371/journal.pbio.3003447)
Supplement: S5 Table — (DOCX) [file pbio.3003447.s015.docx]

**S5 Table. Details of the all-atomistic molecular dynamics simulations, related to Figure 3.**

| System Name | | GPR174-G_s_ (Control) | GPR174-G_s_ (Wat-Rm) | GPR174-G_i_ | P2Y_1_R-G_s_ |
| --- | --- | --- | --- | --- | --- |
| System size | | 12.3×12.3×16.9 nm^3^ | 12.3×12.3×16.9 nm^3^ | 12.3×12.3×15.9 nm^3^ | 12.8×12.8×16.9 nm^3^ |
| Number of Lipids  (Added by CHARMM-GUI) | Cholesterol | 133 | 133 | 134 | 144 |
|  | POPC | 169 | 169 | 170 | 183 |
|  | POPS | 48 | 48 | 48 | 52 |
|  | POPE | 60 | 60 | 60 | 65 |
|  | PSM | 85 | 85 | 86 | 92 |
| Total Number of Lipids | | 495 | 495 | 498 | 536 |
| Number of Waters | | 57,257 | 57,231 | 52,291 | 62,333 |
| Ions | Na^+^ | 199 | 200 | 190 | 213 |
|  | Cl^-^ | 155 | 156 | 141 | 170 |
