# Supplementary material for: Structured water molecules drive activation and G protein selectivity in the GPR174 receptor
Source: PLoS Biol. 2026 May 7;24(5):e3003447. doi: 10.1371/journal.pbio.3003447 (PMC13152116; doi:10.1371/journal.pbio.3003447)
Supplement: S6 Table — (DOCX) [file pbio.3003447.s016.docx]

**S6 Table. The hydrogen-bond residence time (ns) of the hydration-mediated interactions for the GPR174-G_s_ (Control System) and GPR174-G_s_ (Wat-Rm), related to Figure 2.**

| **GPR174-G_s_ (Control System)** | | | | | **GPR174-G_s_ (Wat-Rm)** | | | | |
| --- | --- | --- | --- | --- | --- | --- | --- | --- | --- |
| **Interaction**  **Details** | **Residence Time (ns)** | | | | **Interaction**  **Details** | **Residence Time (ns)** | | | |
|  | **Traj-1** | **Traj-2** | **Traj-3** | **Avg.** |  | **Traj-1** | **Traj-2** | **Traj-3** | **Avg.** |
| W_S1_:Q68@OE1 | 0.226 | 0.157 | 0.158 | 0.18 | W_S1_:Q68@OE1 | 0.159 | 0.201 | 0.189 | 0.18 |
| W_S1_:Q68@NE2 | 0.227 | 0.180 | 0.170 | 0.19 | W_S1_:Q68@NE2 | 0.176 | 0.245 | 0.217 | 0.21 |
| W_S1_:W_S2_ | 0.266 | 0.276 | 0.278 | 0.27 | W_S1_:W_S2_ | 0.273 | 0.286 | 0.293 | 0.28 |
| W_S2_:D65 | 0.335 | 0.298 | 0.297 | 0.31 | W_S2_:D65 | 0.480 | 0.335 | 0.389 | 0.40 |
| W_S2_:D288 | 0.343 | 0.315 | 0.296 | 0.32 | W_S2_:D288 | 0.462 | 0.351 | 0.419 | 0.41 |
| W_S2_:N284@ND2 | 0.316 | 0.235 | 0.247 | 0.27 | W_S2_:N284@ND2 | 0.353 | 0.258 | 0.319 | 0.31 |
| W_S2_:S105@OG | 0.319 | 0.272 | 0.244 | 0.28 | W_S2_:S105@OG | 0.385 | 0.326 | 0.353 | 0.36 |
| W_S3_:N284@OD1 | 0.257 | 0.259 | 0.603 | 0.37 | W_S3_:N284@OD1 | 0.206 | 0.241 | 0.343 | 0.26 |
| W_S3_:D288 | 0.454 | 0.432 | 0.926 | 0.60 | W_S3_:D288 | 0.407 | 0.334 | 0.557 | 0.43 |
| W_S3_:W_S4_ | 0.603 | 0.638 | 0.795 | 0.68 | W_S3_:W_S4_ | 0.291 | 0.504 | 0.522 | 0.44 |
| W_S4_:Y292@OH | 0.393 | 0.406 | 0.679 | 0.49 | W_S4_:Y292@OH | 0.172 | 0.207 | 0.343 | 0.24 |
| W_S5_:Y292@OH | 0.513 | 0.684 | 0.869 | 0.69 | W_S5_:Y292@OH | 0.215 | 0.299 | 0.448 | 0.32 |
| W_S5_:W_S6_ | 0.514 | 0.466 | 0.587 | 0.52 | W_S5_:W_S6_ | 0.462 | 0.422 | 0.533 | 0.47 |
| W_S6_:R116 | 0.752 | 0.822 | 1.042 | 0.87 | W_S6_:R116 | 1.476 | 0.627 | 0.781 | 0.96 |
| W_S6_:W_S7_ | 0.529 | 0.482 | 0.573 | 0.53 | W_S6_:W_S7_ | 0.242 | 0.493 | 0.528 | 0.42 |
| W_S7_:T205@OG1 | 0.837 | 0.722 | 0.957 | 0.84 | W_S7_:T205@OG1 | 0.170 | 0.565 | 0.719 | 0.49 |
| W_S7_:W_S8_ | 0.508 | 0.471 | 0.509 | 0.50 | W_S7_:W_S8_ | 0.150 | 0.505 | 0.580 | 0.41 |
| W_S8_:T208@OG1 | 0.776 | 1.332 | 1.917 | 1.34 | W_S8_:T208@OG1 | 0.357 | 0.967 | 1.328 | 0.88 |
| W_G1_:R116 | 0.421 | 0.373 | 0.653 | 0.48 | W_G1_:R116 | 0.634 | 0.267 | 0.578 | 0.49 |
| W_G1_:Y391^Gα^@O | 0.417 | 0.375 | 0.653 | 0.48 | W_G1_:Y391^Gα^@O | 0.629 | 0.265 | 0.577 | 0.49 |
| W_G1_:E392^Gα^@O | 0.430 | 0.377 | 0.666 | 0.49 | W_G1_:E392^Gα^@O | 0.628 | 0.271 | 0.577 | 0.49 |
| E392^Gα^@OE1:N297@N | 2.032 | 1.413 | 2.093 | 1.85 | E392^Gα^@OE1:N297@N | 0.806 | 1.146 | 1.538 | 1.16 |
| E392^Gα^@OE2:N297@N | 2.482 | 1.563 | 1.643 | 1.90 | E392^Gα^@OE2:N297@N | 0.784 | 1.000 | 0.928 | 0.90 |
