# Supplementary material for: Structured water molecules drive activation and G protein selectivity in the GPR174 receptor
Source: PLoS Biol. 2026 May 7;24(5):e3003447. doi: 10.1371/journal.pbio.3003447 (PMC13152116; doi:10.1371/journal.pbio.3003447)
Supplement: S7 Table — (DOCX) [file pbio.3003447.s017.docx]

**S7 Table. The hydrogen-bond residence time (ns) of the hydration-mediated interactions for the GPR174-G_s_ and GPR174-G_i_ complexes, related to Figure 2.**

| **GPR174-G_s_** | | | | | **GPR174-G_i_** | | | | |
| --- | --- | --- | --- | --- | --- | --- | --- | --- | --- |
| **Interaction**  **Details** | **Residence Time (ns)** | | | | **Interaction**  **Details** | **Residence Time (ns)** | | | |
|  | **Traj-1** | **Traj-2** | **Traj-3** | **Avg.** |  | **Traj-1** | **Traj-2** | **Traj-3** | **Avg.** |
| W_S1_:Q68@OE1 | 0.226 | 0.157 | 0.158 | 0.18 | W_S1_:Q68@OE1 | 0.106 | 0.160 | 0.136 | 0.13 |
| W_S1_:Q68@NE2 | 0.227 | 0.180 | 0.170 | 0.19 | W_S1_:Q68@NE2 | 0.107 | 0.168 | 0.130 | 0.13 |
| W_S1_:W_S2_ | 0.266 | 0.276 | 0.278 | 0.27 | W_S1_:W_S2_ | 0.257 | 0.263 | 0.264 | 0.26 |
| W_S2_:D65 | 0.335 | 0.298 | 0.297 | 0.31 | W_S2_:D65 | 0.113 | 0.177 | 0.152 | 0.14 |
| W_S2_:D288 | 0.343 | 0.315 | 0.296 | 0.32 | W_S2_:D288 | 0.112 | 0.163 | 0.153 | 0.14 |
| W_S2_:N284@ND2 | 0.316 | 0.235 | 0.247 | 0.27 | W_S2_:N284@ND2 | 0.109 | 0.160 | 0.139 | 0.13 |
| W_S2_:S105@OG | 0.319 | 0.272 | 0.244 | 0.28 | W_S2_:S105@OG | 0.111 | 0.171 | 0.146 | 0.14 |
| W_S3_:N284@OD1 | 0.257 | 0.259 | 0.603 | 0.37 | W_S3_:N284@OD1 | 0.120 | 0.127 | 0.112 | 0.12 |
| W_S3_:D288 | 0.454 | 0.432 | 0.926 | 0.60 | W_S3_:D288 | 0.134 | 0.140 | 0.118 | 0.13 |
| W_S3_:W_S4_ | 0.603 | 0.638 | 0.795 | 0.68 | W_S3_:W_S4_ | 0.728 | 0.578 | 0.544 | 0.62 |
| W_S4_:Y292@OH | 0.393 | 0.406 | 0.679 | 0.49 | W_S4_:Y292@OH | 0.117 | 0.114 | 0.109 | 0.11 |
| W_S5_:Y292@OH | 0.513 | 0.684 | 0.869 | 0.69 | W_S5_:Y292@OH | 0.185 | 0.196 | 0.158 | 0.18 |
| W_S5_:W_S6_ | 0.514 | 0.466 | 0.587 | 0.52 | W_S5_:W_S6_ | 0.407 | 0.457 | 0.439 | 0.43 |
| W_S6_:R116 | 0.752 | 0.822 | 1.042 | 0.87 | W_S6_:R116 | 0.419 | 0.158 | 0.194 | 0.26 |
| W_S6_:W_S7_ | 0.529 | 0.482 | 0.573 | 0.53 | W_S6_:W_S7_ | 0.366 | 0.347 | 0.422 | 0.38 |
| W_S7_:T205@OG1 | 0.837 | 0.722 | 0.957 | 0.84 | W_S7_:T205@OG1 | 0.196 | 0.160 | 0.178 | 0.18 |
| W_S7_:W_S8_ | 0.508 | 0.471 | 0.509 | 0.50 | W_S7_:W_S8_ | 0.301 | 0.317 | 0.403 | 0.34 |
| W_S8_:T208@OG1 | 0.776 | 1.332 | 1.917 | 1.34 | W_S8_:T208@OG1 | 0.517 | 0.180 | 0.290 | 0.33 |
| W_G1_:R116 | 0.421 | 0.373 | 0.653 | 0.48 | W_G1_:R116 | 0.081 | 0.095 | 0.107 | 0.09 |
| W_G1_:Y391^Gα^@O | 0.417 | 0.375 | 0.653 | 0.48 | W_G1_:C351^Gα^@O | 0.151 | 0.105 | 0.107 | 0.12 |
| W_G1_:E392^Gα^@O | 0.430 | 0.377 | 0.666 | 0.49 | W_G1_:G352^Gα^@O | 0.111 | 0.099 | 0.097 | 0.10 |
