# Supplementary material for: Structured water molecules drive activation and G protein selectivity in the GPR174 receptor
Source: PLoS Biol. 2026 May 7;24(5):e3003447. doi: 10.1371/journal.pbio.3003447 (PMC13152116; doi:10.1371/journal.pbio.3003447)
Supplement: S9 Table — (DOCX) [file pbio.3003447.s019.docx]

**S9 Table. Cell-surface expression levels of GPR174 hydration-network mutants in the G_i_ signaling assay, determined by ELISA, related to Figure 2.**

| Mutation | Expression ± SEM (% WT) | Sample size |
| --- | --- | --- |
| WT | 100±2 | 6 |
| D65^2.50^N | 101±3 | 3 |
| Q68^2.53^L | 98±6 | 3 |
| S105^3.39^A | 105±8 | 3 |
| T205^5.58^V | 117±15 | 3 |
| T208^5.61^V | 107±9 | 3 |
| N284^7.45^L | 104±19 | 3 |
| D288^7.49^N | 85±9 | 3 |
| Y292^7.53^F | 110±8 | 3 |

Expression values are shown as mean ± SEM and normalized to WT (% WT). Sample size (n) indicates the number of independent experiments. Data are shown as mean ± SEM from at least three independent experiments, each performed in triplicate.
