# Supplementary material for: Structured water molecules drive activation and G protein selectivity in the GPR174 receptor
Source: PLoS Biol. 2026 May 7;24(5):e3003447. doi: 10.1371/journal.pbio.3003447 (PMC13152116; doi:10.1371/journal.pbio.3003447)
Supplement: S10 Table — (DOCX) [file pbio.3003447.s020.docx]

**S10 Table. Hydration cavity volumes in active-state class A GPCRs, related to Figure 3.**

| Receptor | CWC (Å³) | JWC (Å³) | EWC (Å³) | Coupling (GtP) |
| --- | --- | --- | --- | --- |
| GPR174 | 43.6 | 69.7 | 155.8 | G_s_, G_i/o_* |
| P2Y_10_R | 40.1 | 59.4 | 119.1 | G_i/o_ |
| GPR55 | 89.3 | 33.9 | 111.9 | G_12/13_ |
| PF_2_αR | 29.6 | 45.6 | 93.0 | G_q/11_, G_s_ |
| GPR20 | 49.5 | 41.1 | 92.8 | G_i/o_ |
| H_4_R | 59.4 | 35.6 | 88.1 | G_i/o_, G_q/11_ |
| GAL_2_R | 47.8 | 30.8 | 47.0 | G_q/11_, G_i/o_, G_12/13_ |
| β_2_AR | 24.8 | 48.9 | 22.7 | G_s_, G_i/o_* |
| P2Y_1_R | 23.3 | 38.0 | 33.4 | G_q/11_, G_i/o_ |
| GPR52 | 75.4 | 27.9 | 35.9 | G_s_ |
| FFA_2_R | 38.4 | 58.8 | 30.1 | G_q/11_, G_i/o_ |
| GPR119 | 39.3 | 37.9 | 7.5 | G_s_ |

Reported coupling annotations were compiled from the IUPHAR/BPS Guide to Pharmacology database (GtoPdb). Receptors with reported coupling to both G_s_-family and G_i/o_-family pathways are marked with *.
