# Supplementary material for: Structured water molecules drive activation and G protein selectivity in the GPR174 receptor
Source: PLoS Biol. 2026 May 7;24(5):e3003447. doi: 10.1371/journal.pbio.3003447 (PMC13152116; doi:10.1371/journal.pbio.3003447)
Supplement: S12 Table — (DOCX) [file pbio.3003447.s022.docx]

**S12 Table. Cell-surface expression levels of wild-type and mutant P2Y_1_R, determined by ELISA, related to Figure 3.**

| Mutation | Expression ± SEM (% WT) | Sample size |
| --- | --- | --- |
| WT | 100±6 | 5 |
| D97^2.50^N | 92±17 | 3 |
| S138^3.39^A | 77±4 | 3 |
| R149^3.50^Q | 126±10 | 3 |
| Y214^5.35^F | 100±4 | 3 |
| Y303^7.32^F | 88±3 | 3 |
| R310^7.39^Q | 100±3 | 3 |
| D320^7.49^N | 112±4 | 3 |
| Y324^7.53^F | 102±3 | 3 |

Expression values are shown as mean ± SEM and normalized to WT (% WT). Sample size (n) indicates the number of independent experiments. Data are shown as mean ± SEM from at least three independent experiments, each performed in triplicate.
