# Supplementary material for: Structured water molecules drive activation and G protein selectivity in the GPR174 receptor
Source: PLoS Biol. 2026 May 7;24(5):e3003447. doi: 10.1371/journal.pbio.3003447 (PMC13152116; doi:10.1371/journal.pbio.3003447)
Supplement: S13 Table — (DOCX) [file pbio.3003447.s023.docx]

**S13 Table.** **Cell surface expression of wild-type and mutant GPR174 co-expressed with G_s_,** **related to Figures 2 and 4.**

| Mutation | Expression ± SEM (% WT) | Sample size |
| --- | --- | --- |
| WT | 100±3 | 6 |
| R53^ICL1^A | 128±5 | 3 |
| V55^2.40^A | 52±0 | 3 |
| V55^2.40^F | 138±2 | 3 |
| F57^2.42^A | 62±1 | 3 |
| M58^2.43^A | 80±1 | 3 |
| M58^2.43^F | 113±4 | 3 |
| D65^2.50^N | 99±2 | 3 |
| R75^2.60^A | 131±5 | 3 |
| Y79^2.64^A | 100±5 | 3 |
| Q68^2.53^L | 184±6 | 3 |
| Y99^3.33^A | 102±9 | 3 |
| S105^3.39^A | 127±2 | 3 |
| I112^3.46^A | 70±1 | 3 |
| R115^3.49^A | 110±1 | 3 |
| R115^3.49^Q | 79±1 | 3 |
| R116^3.50^A | 113±4 | 3 |
| R116^3.50^Q | 109±3 | 3 |
| L120^3.54^A | 134±0 | 3 |
| D128^ICL2^A | 132±5 | 3 |
| C129^ICL2^A | 110±2 | 3 |
| F152^4.60^A | 206±8 | 3 |
| R156^4.64^A | 142±4 | 3 |
| F169^ECL2^A | 53±3 | 3 |
| T205^5.58^V | 135±2 | 3 |
| T208^5.61^V | 118±1 | 3 |
| M218^ICL3^A | 116±2 | 3 |
| D221^ICL3^A | 116±3 | 3 |
| K225^6.30^A | 122±1 | 3 |
| Y246^6.51^A | 124±5 | 3 |
| F250^6.55^A | 159±7 | 3 |
| K257^6.62^A | 135±6 | 3 |
| N284^7.45^L | 100±1 | 3 |
| D288^7.49^N | 131±7 | 3 |
| Y292^7.53^F | 147±2 | 3 |

Data are shown as mean ± SEM from at least three independent experiments, each performed in triplicate.
