# Supplementary material for: Structured water molecules drive activation and G protein selectivity in the GPR174 receptor
Source: PLoS Biol. 2026 May 7;24(5):e3003447. doi: 10.1371/journal.pbio.3003447 (PMC13152116; doi:10.1371/journal.pbio.3003447)
Supplement: S14 Table — (DOCX) [file pbio.3003447.s024.docx]

**S14 Table. GPR174-induced G_s_ dissociation assays of wild-type and mutant GPR174, related to Figure 4.**

| Mutation | pEC_50_ ± SEM | Span ± SEM | Sample size |
| --- | --- | --- | --- |
| WT | 6.496±0.141 | -0.100±0.004 | 5 |
| R53^ICL1^A | NA | NA | 3 |
| V55^2.40^A | 6.730±0.597 | -0.043±0.014 | 3 |
| V55^2.40^F | NA | NA | 3 |
| F57^2.42^A | NA | NA | 3 |
| M58^2.43^A | NA | NA | 3 |
| M58^2.43^F | NA | NA | 3 |
| R75^2.60^A | NA | NA | 3 |
| Y79^2.64^A | NA | NA | 3 |
| Y99^3.33^A | NA | NA | 3 |
| I112^3.46^A | NA | NA | 3 |
| R115^3.49^A | NA | NA | 4 |
| R115^3.49^Q | NA | NA | 6 |
| R116^3.50^A | NA | NA | 3 |
| L120^3.54^A | 5.951±0.075 | -0.095±0.010 | 3 |
| D128^ICL2^A | 5.786±0.065 | -0.083±0.021 | 3 |
| C129^ICL2^A | 5.884±0.464 | -0.079±0.007 | 3 |
| F152^4.60^A | NA | NA | 3 |
| R156^4.64^A | NA | NA | 3 |
| F169^ECL2^A | NA | NA | 3 |
| M218^ICL3^A | 6.570±0.899 | -0.043±0.010 | 3 |
| D221^ICL3^A | 5.940±0.564 | -0.067±0.009 | 3 |
| K225^6.30^A | NA | NA | 3 |
| Y246^6.51^A | NA | NA | 3 |
| F250^6.55^A | NA | NA | 3 |
| K257^6.62^A | NA | NA | 3 |

Data were analyzed using a three-parameter logistic equation to determine pEC_50_ and Span. All data were normalized to the response at the lowest agonist concentration. Data are presented as mean ± SEM from at least three independent experiments, each performed in triplicate. Values in S1 Data are shown as the mean of triplicates for each independent experiment. NA, not applicable.
