# Supplementary material for: Structured water molecules drive activation and G protein selectivity in the GPR174 receptor
Source: PLoS Biol. 2026 May 7;24(5):e3003447. doi: 10.1371/journal.pbio.3003447 (PMC13152116; doi:10.1371/journal.pbio.3003447)
Supplement: S16 Table — (DOCX) [file pbio.3003447.s026.docx]

**S16 Table. Cell surface expression of wild-type and mutant GPR174 co-expressed with G_i_, related to Figure 4.**

| Mutation | Expression ± SEM (% WT) | Sample size |
| --- | --- | --- |
| WT | 100±2 | 6 |
| R53^ICL1^A | 145±8 | 5 |
| V55^2.40^A | 54±1 | 3 |
| V55^2.40^F | 123±4 | 5 |
| F57^2.42^A | 67±2 | 3 |
| M58^2.43^A | 73±1 | 3 |
| M58^2.43^F | 98±9 | 5 |
| R75^2.60^A | 190±28 | 4 |
| Y79^2.64^A | 121±12 | 4 |
| Y99^3.33^A | 91±5 | 3 |
| I112^3.46^A | 78±1 | 3 |
| R115^3.49^A | 117±3 | 3 |
| R115^3.49^Q | 81±3 | 3 |
| R116^3.50^A | 133±15 | 5 |
| R116^3.50^Q | 117±8 | 5 |
| L120^3.54^A | 133±4 | 3 |
| P123^ICL2^A | 90±3 | 3 |
| D128^ICL2^A | 153±17 | 4 |
| C129^ICL2^A | 114±4 | 3 |
| F152^4.60^A | 173±3 | 3 |
| R156^4.64^A | 162±21 | 5 |
| F169^ECL2^A | 73±7 | 3 |
| M218^ICL3^A | 127±3 | 3 |
| D221^ICL3^A | 170±23 | 4 |
| E224^6.29^A | 94±1 | 3 |
| K225^6.30^A | 112±4 | 3 |
| Y246^6.51^A | 94±20 | 3 |
| F250^6.55^A | 115±17 | 3 |
| K257^6.62^A | 119±30 | 3 |

Data are shown as mean ± SEM from at least three independent experiments, each performed in triplicate.
