# Supplementary material for: Structured water molecules drive activation and G protein selectivity in the GPR174 receptor
Source: PLoS Biol. 2026 May 7;24(5):e3003447. doi: 10.1371/journal.pbio.3003447 (PMC13152116; doi:10.1371/journal.pbio.3003447)

Figure S1B. Original uncropped SDS-PAGE image corresponding to the analysis of the purified GPR174-G_s complex

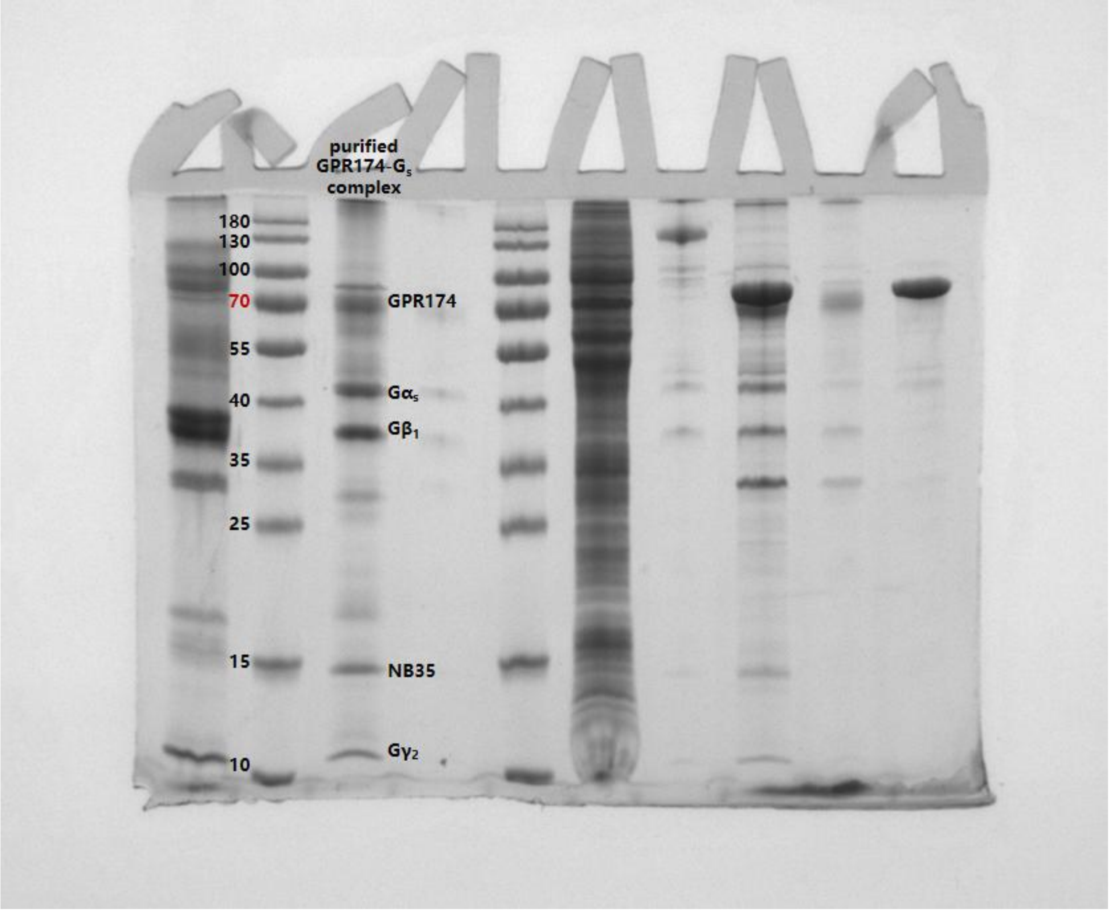

Figure S2B. Original uncropped SDS-PAGE image corresponding to the analysis of the purified GPR174-G_i complex

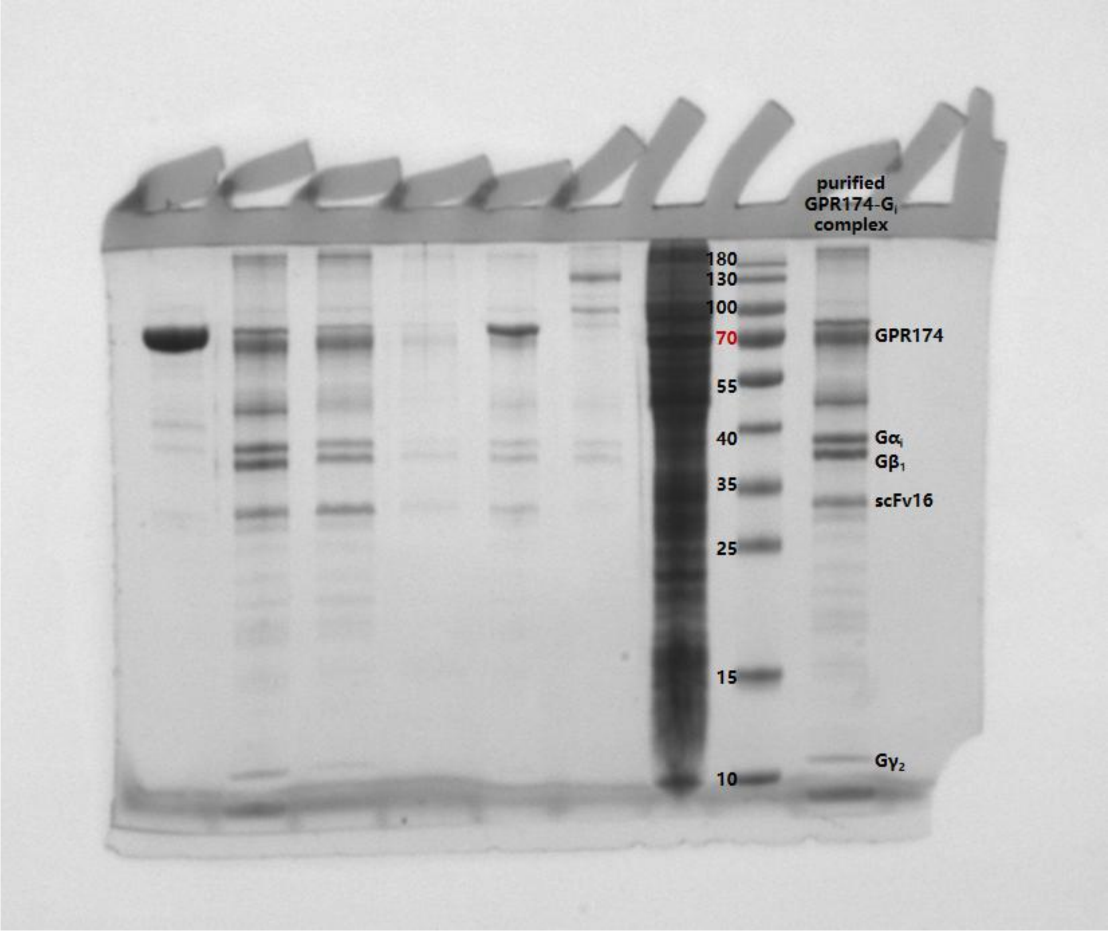

Supplement: S1 Raw Images — (PDF) [file pbio.3003447.s028.pdf]
